# The library of isolated bacteria from gut microbiota in classical fish models: Zebrafish (*Danio rerio*), marine (*Oryzias melastigma*) and freshwater (*Oryzias latipes*) medaka

**DOI:** 10.1371/journal.pone.0347661

**Published:** 2026-05-15

**Authors:** Lan-Chen Zhang, Yan Li, Ming-Fei Wu, Jin-Qiu Jia, Pan-Pan Jia

**Affiliations:** School of Public Health, Chongqing Medical University, Chongqing, China; Karlsruhe Institute of Technology: Karlsruher Institut fur Technologie, GERMANY

## Abstract

Zebrafish (*Danio rerio*), Marine medaka (*Oryzias melastigma*), and freshwater medaka (*Oryzias latipes*) are three major animal species in evaluation of environmental pollutant toxicity and building of human disease models with conserved physiological and molecular pathways. Bacterial strains from gut microbiota should firstly be isolated for their functions’ exploration. In this study, we optimized a culture-based workflow under aerobic and anaerobic conditions to recover intestinal bacteria from adult zebrafish (ZF), marine medaka (MM), and freshwater medaka (OL) using both dissection-based and *in vivo* sampling. The isolates were then identified by Gram staining, 16S rRNA gene sequencing, phylogenetic analysis, and API 20E-based biochemical characterization. The culturable bacteria isolated from gut microbiota were identified belonged to 14 genera in ZF, 12 genera in MM, and 18 genera in OL. Comparative analysis showed clear differences in the composition of cultured gut bacteria among the three fish species. Overall, the library and the differences of gut bacteria in three key fish models provided the potential applications of bacterial strains as the probiotics, to against the fish pathogens and to increase the pollutant toxicity-resistant. Moreover, the bacterial library will support the deep researches combined with germ-free (GF) animals to clarify the relationships of intestinal microbiota to host health.

## 1. Introduction

Zebrafish (*Danio rerio*), marine medaka (*Oryzias melastigma*) and freshwater medaka (*Oryzias latipes*) are useful and sensitive models for monitoring various pollutants in fresh water, ocean and estuary environments including heavy metals, endocrine disruptors, antibiotics, and pathogens [1–3]. Among these fish, zebrafish (ZF) were considered as the key vertebrate models for understanding the role of gut microbiota in the developing intestinal tract and enteric nervous system interactions [4, 5]. Two typical models of medaka included the marine medaka (MM) and freshwater medaka (OL) in the family Adrianichthyidae, which have been extensively used worldwide in evaluation of pollutants’ toxicity and inner mechanisms with the omics analyses and genomic sequencing [6–8], providing an important experimental basis for their use in the present study. Moreover, fish had a more abundant of gut microbiota from early life to adult stages [9, 10]. However, the gut microbiota of fish models that can be isolated, as well as their special functions, remain incompletely discovered. In fact, the live environments and the kinds of food will hugely influence the community of gut microbiota, which maybe contribute to differences between individuals and laboratories. Beyond doubt, the microbial community of ZF and medaka especially the marine species will dramatically distinct for their live habits. Thus, the culturable microbial profile of fish will be significant for understanding intestinal bacterial strains and their functions in host health.

The ZF and medaka model has advantages of easy breeding in the laboratory with small size, short generation (2–3 months), high spawning ability, transparent embryos/larvae, identified genome, and human disease models, *etc* [1,6,11,12]. Thus, the interactions between microbes and hosts can be studied by analyzing the genotypes and community competition of specific microbes, as well as how microbial colonization or metabolic products affect the organism biological processes [13,14]. The microbiota of vertebrate plays key roles in host development, which can affect the immune system, provide nutrients, and stimulate intestinal epithelium differentiation [15,16]. In zebrafish, the culturable gut microbiota diversity and communities assemble in tandem with host development, and spatial and temporal species-richness relationships in microbial assembly and dynamics during life shaped [17,18]. However, the profile of isolated gut microbiota of zebrafish and marine and freshwater medaka has not been sufficiently discovered. Zebrafish and medaka can be matured within approximately 2–3 months [19–21], and adult fish are generally considered to harbor a more stable gut microbiota [22,23], making them suitable for gut bacterial isolation and comparative analysis. The culture researches on environmental and gut bacteria were conducted on water and soil samples, adult shrimps and fish, to screen the potential antibiotic and heavy-metal resistance strains, such as *Aeromonas* sp., and *Vibrio* sp. [24,25].

The isolation of gut bacteria from fish models can provide the culturable strains, among which can be selected as candidate probiotics beneficial for aquaculture [26]. For example, the bacterial isolates from freshwater fish (*Labeo rohita*) gut, including the *Lactobacillus plantarum* VSG3, *Pseudomonas aeruginosa* VSG2, and *Bacillus subtilis* VSG1, showed the potential probiotics’ characteristics [27]. Several bacteria in marine fish species are still need further study to reveal their response to environmental factors and pathogens form the antimicrobial peptides, probiotics, and medicinal treatment [28]. The intestinal microbiota of animals, including fish models, is considered as a critical indicator of environmental and organism health, and the various diets of laboratory and surroundings influence the organism growth and fecundity in turn [13,29]. Despite increasing interest in fish gut microbiota, comparative culturable resources for intestinal bacterial isolates from zebrafish, marine medaka, and freshwater medaka, and the functional characterization of such isolates are still insufficient. To address this gap, in this study, we comprehensively isolated and identified the strains of three typical fish models *via* different sampling methods and characteristics detections. The natural composition of intestinal microbiota and culturable bacterial strains of these models were revealed and compared, as well as the differences between the fish species and live conditions. Importantly, the prospective applications for host health were proposed based on the analyses of key functions of the intestinal bacterial strains from fish models. Finally, the library of isolated fish bacteria was generated firstly under aerobic and anaerobic conditions supplying with gram-positive and negative detections and metabolic functions assay, which provide the source to select the probiotic intestinal bacteria on special disease therapy and health regulation.

## 2. Methods and materials

### 2.1. Fish maintenance and sampling of the gut microbiota

Wild type zebrafish, marine medaka, and freshwater medaka were obtained from the Institute of Hydrobiology, Chinese Academy of Sciences, the South China Sea Institute of Oceanology, Chinese Academy of Sciences, and cultured with the following standard procedure in our laboratory. The zebrafish and *O. latipes* were maintained in auto-system with the filtered pure water and were fed with newly hatched brine shrimp (*Artemia* sp*.*) twice daily, with a temperature of 28 ± 2.0℃ and a 14:10 h light/dark cycle. While, the marine medaka were maintained in glass tanks with the culture seawater refreshed every 48 h and were fed with newly hatched brine shrimp twice daily and the same conditions with above. The circulating seawater was prepared with ultra-pure water and activated salt, with a final salinity of 35‰, and then was UV-sterilized before use. The fertilized eggs of medaka were collected daily from the tank bottom and could be cultured for 3 months to be adult. At adult, three individuals per fish species was randomly selected and washed with ultrapure water, anesthetized with 100 mg/L MS-222, and then dissected for intestine sampling. In parallel, three additional individuals per fish species was randomly selected for *in vivo* intestinal sampling. Next, the samples from three different fish species were immediately cultured *in vitro* with prepared plates and liquid medium for isolation and purification of microbial community. Meanwhile, three fish of each species were collected for accurate 16S rRNA gene absolute quantification sequencing of bacteria.

### 2.2. Chemicals and preparation

In this study, the NaCl (CAS: 7647-14-5), soy peptone (Coolaber, Cat#CS10383), tryptone (Oxoid, Cat#LP0042), and agar powder (Solarbio, CAS: 9002-18-0) were used to prepare the tryptic soy agar (TSA) plates and tryptic soy broth (TSB) medium, and the sterile defibrinated sheep blood (Solarbio, Cat#TX0030) was added into TSA to prepare the 5% blood plates. The brain heat infusion (BHI, Cat#B8130) medium, glycerol (>99.0%, CAS: 56-81-5, Cat#G8190), and the bacterial genomic DNA extraction kit (Cat#D1600) were purchased form the Solarbio company. The Taq^TM^ (TaKaRa, Cat#R001A) kits and the bacterial genomic DNA extraction kit (Solarbio, Cat#D1600) were used for the normal PCR assay to identify the bacterial strain and GF model samples. In addition, the 3-Aminobenzoic acid ethyl ester or methane-sulfonate salt (MS-222, 98%) was obtained from Aladdin (Los Angeles, California, USA). All other chemical reagents used in this study were of analytical grade.

### 2.3. Isolation and purification of intestinal bacteria in adult fish models

**Fig 1:**
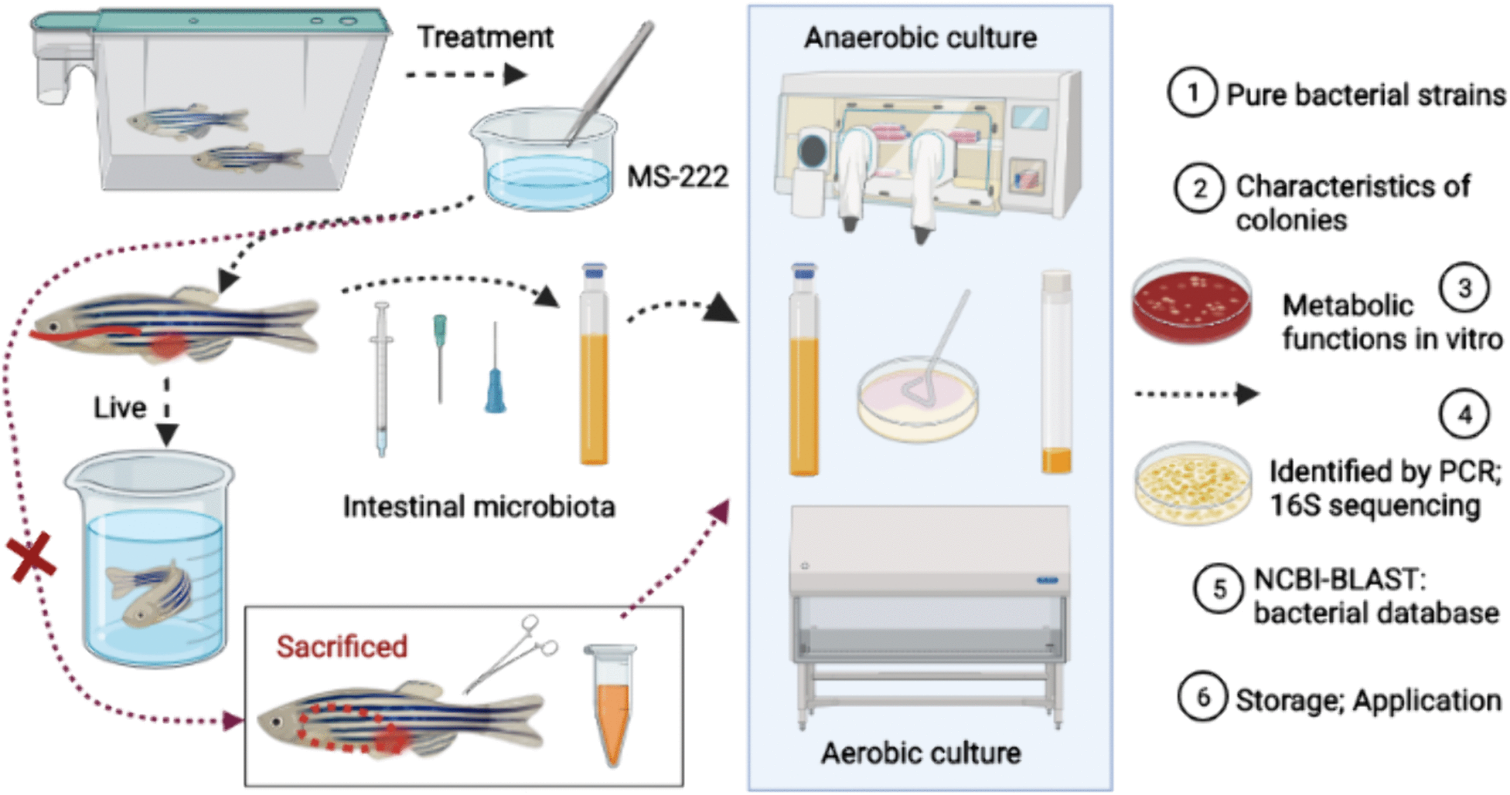
Flow chart of isolation and identification of intestinal bacteria from typical adult fish models. The progress involved the treatment of adult fish models (zebrafish as the example) with washing and anaesthetized in MS-222, sacrificed to dissect the gut tissues, then culture the gut microbiota in the TSB and BHI medium, selected distinct bacterial colonies and purify the strains on TSA and Blood plates, identify the strains by PCR and 16S rRNA gene sequencing as well as the BLASTn search, finally store the bacteria and submitted the information on NCBI database.

With optimized approaches in this study and based on the methods used in previous and our studies [13,18,22,30], the possible culturable intestinal bacteria form fish models were isolated and identified with the colony characteristics and metabolic functions. Bacterial isolation was performed in a clean bench based on optimized procedures from previous studies and our preliminary experiments. Adult fish were first rinsed three times with sterile water and anesthetized with 100 mg/L MS-222. The body surface was then sterilized with 75% ethanol and rinsed again with sterile water. For dissection-based sampling, intact intestines were aseptically removed and divided into anterior, middle, and posterior sections. For *in vivo* sampling, intestinal contents were collected directly from anesthetized fish using a modified gavage needle. The intestinal contents were suspended in sterile medium, and the supernatant was inoculated onto TSA, 5% blood agar, TSB, and BHI media under both aerobic and anaerobic conditions. All plates were incubated at 30 °C in a biochemical incubator. Distinct colonies were selected according to morphological characteristics, including size, color, surface appearance, and colony margin, and were repeatedly subcultured for 5–6 generations until pure isolates were obtained. Purified colonies were then inoculated into TSB broth and cultured at 30 °C with shaking at 150 rpm for 24–48 h for expansion. Isolates were finally preserved in 30% glycerol at −80 °C for subsequent identification and in vitro assays. In some cases, the original culture plates were sealed under sterile conditions and stored at 4 °C for up to 2 weeks. Additional late-emerging colonies appearing during this period were further picked and purified as supplementary isolates (Fig 1).

### 2.4. Identification of intestinal bacteria in adult fish models

#### 2.4.1. Gram staining.

Gram staining was performed for preliminary phenotypic characterization of purified isolates. Fresh bacterial cultures were prepared in TSB at 30 °C with shaking for 16–20 h. Bacterial smears were prepared on glass slides, heat-fixed, and stained using the standard crystal violet–iodine–decolorization–safranin procedure. After air drying, stained cells were examined under an oil immersion lens. Isolates showing bluish-purple staining were recorded as Gram-positive, whereas those showing pinkish-red staining were recorded as Gram-negative.

#### 2.4.2. Genomic DNA extraction, 16S rRNA gene amplification, and taxonomic assignment.

Genomic DNA was extracted from purified bacterial isolates using a bacterial genomic DNA extraction kit (Solarbio, Cat#D1600) according to the manufacturer’s instructions. DNA quality and concentration were assessed before PCR amplification. The nearly full-length 16S rRNA gene was amplified using the universal bacterial primers 27F and 1492R. PCR products were checked by agarose gel electrophoresis and subsequently sequenced. The resulting sequences were examined and assembled using Chromas and DNAman software, and taxonomic assignment was performed by comparison against the NCBI nucleotide database and the 16S ribosomal RNA database for Bacteria and Archaea using BLAST. The best-matched reference sequences were used for preliminary genus-level identification and construction of the bacterial isolate library.

### 2.5. Composition and phylogeny tree of gut microbiota in adult fish models

According to the sequencing database of bacterial strains from fish models (*Danio rerio*, Marine medaka, *Oryzias latipes*), the composition of culturable microbiota was analyzed and presented with pie chart and relative abundances of each genus in three kinds of fish. More importantly, the microbiota of different fish detected *via* 16S rRNA V3-V4 regions sequencing and similarity analyses were performed based on the blast to the bacterial database in NCBI website. Furthermore, the phylogeny tree of these bacteria was compared to the similar strains according to the sequences downloaded from NCBI database. Additionally, the differences and similarity of development of culturable bacteria in three fish models were discovered and explored the relationships between live environments.

### 2.6. The metabolic functions of fish gut bacteria measured by API 20E kits

Representative isolates from each genus recovered from zebrafish, marine medaka, and freshwater medaka were selected for biochemical characterization using the API 20E system (Ref 20100 Kits, bioMerieux® SA, France) according to the manufacturer’s instructions. Fresh bacterial cultures were prepared in TSB at 30 °C with shaking for 12–18 h and resuspended in sterile 0.85% NaCl before inoculation into the test strips. After incubation for 18–24 h, the reactions were recorded according to the manufacturer’s interpretation criteria. The biochemical traits evaluated included o-nitrophenyl-β-D-galactopyranoside (ONPG), arginine dihydrolase (ADH), lysine decarboxylase (LDC), ornithine decarboxylase (ODC), citrate (CIT), hydrogen sulfide (H_2_S), urease(URE), tryptophan deaminase (TDA), indole test (IND), Voges Proskauer (VP), gelatinase (GEL), glucose (GLU), mannitol (MAN), inositol (INO), sorbitol (SOR), rhamnose (RHA), saccharose (SAC), melibiose (MEL), amygdalin (AMY), and arabinose (ARA), cytochrome oxidase (OX), and nitrogen dioxide (NO_2_). These data were used for preliminary comparison of the metabolic characteristics of representative gut bacterial isolates among the three fish species.

### 2.7. Statistical analysis

The statistical analysis was performed using the Kolmogorov-Smirnov test and Leven’s test to define the normality of data and the homogeneity of variance. The differences between the variables were calculated by t-test and one-way analysis of variance (ANOVA), followed by Dunnett's test using SPSS 20.0 software (SPSS, Chicago, IL, USA). The figures were represented by using the GraphPad Prism 10, with the average data presented as the histogram or pie of each group.

### 2.8. Ethic statement

The experiment protocol was approved by the Animal Care and Use Committee of Chongqing, and the Institutional Animal Care and Use Committee of Chongqing Medical University, Chongqing, China, which was referred to the Administration of Experimental Animals issued by the Ministry of Science and Technology, and standards for experimental animals issued by the State Bureau of Quality and Technical Supervision (Approval ID: GB14922−2001 to GBT14927−2001).

For euthanasia, fish were immersed in 200 mg/L MS-222 for approximately 10–20 min until no opercular movement was observed. Measures taken to minimize animal suffering included the use of adequate anesthesia, gentle handling during *in vivo* sampling, and post-procedural observation of experimental fish.

## 3. Results

### 3.1. Gut microbiota of adult zebrafish, marine medaka, and freshwater medaka

**Fig 2:**
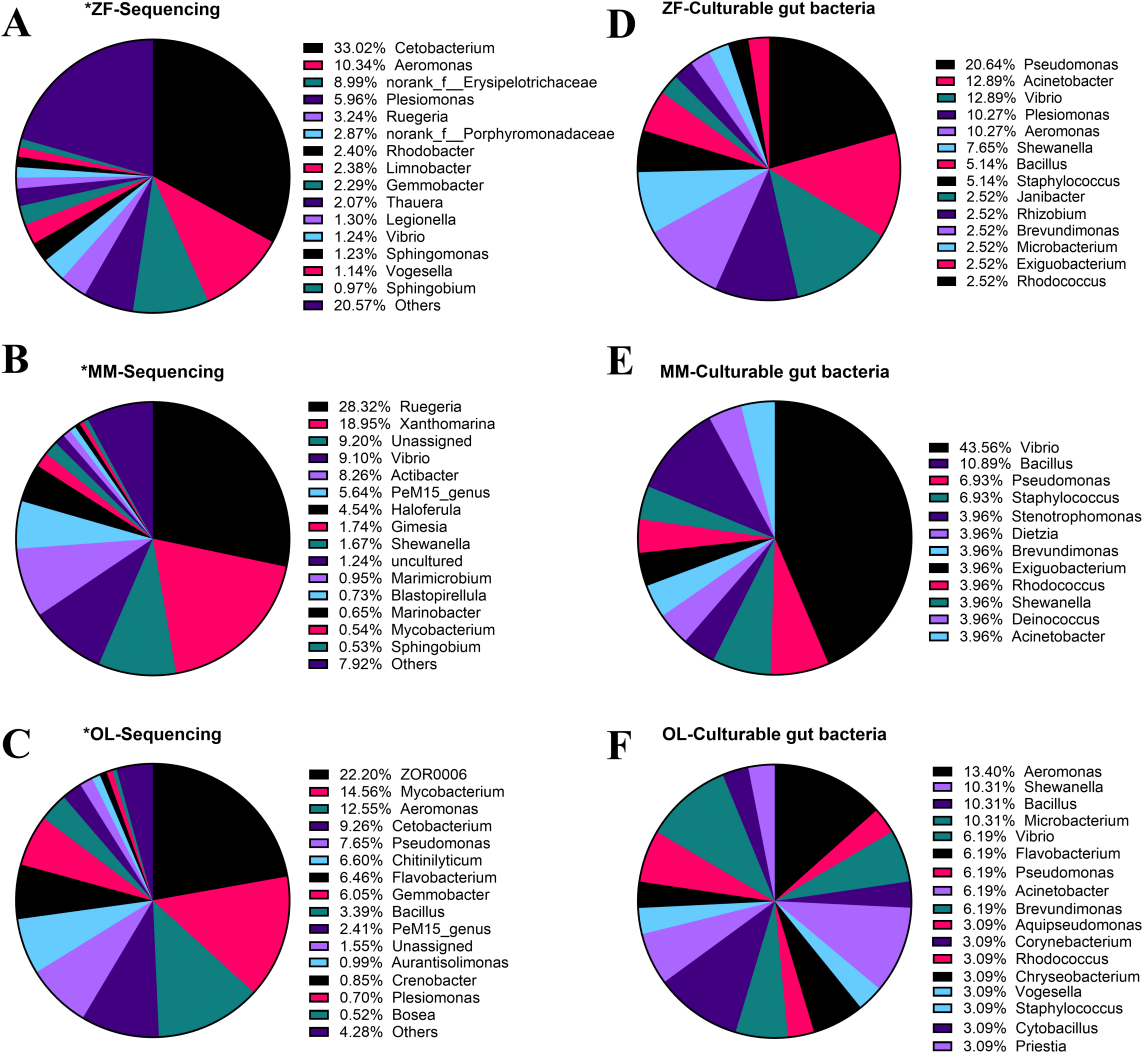
Composition of gut microbiota and isolated bacteria in adult zebrafish, marine medaka and freshwater medaka species. **(A)** The percentage chart of bacterial genera by the sequencing of zebrafish, **(B)** marine medaka, **(C)** freshwater medaka intestinal flora. **(D-F)** Bar chart of the percentage of genera in the zebrafish, marine medaka, freshwater medaka intestinal flora screened out in this experiment.

To explore the microbial composition and functions, the gut microbiota of three classical fish models in laboratory was analyzed in this study. In details, the 16S rRNA high-throughput sequencing data was obtained from our previous studies [23,30–32], and unpublished data, and then the composition of gut microbiota in different fish models was compared (Fig 2). It was found that the zebrafish gut microbiota was composed of 433 bp belonging to 1 Domain, 1 Kingdom, 43 Phylum, 93 Class, 200 Order, 381 Family, 795 Genera, 1318 Species, and 2181 OTUs from a total of 18 samples. Among gut microbiota of zebrafish, the main genera involved the *Cetobacterium* (33.02%), *Aeromonas* (10.34%), *Plesiomonas* (5.96%), *Rhodobacter* (2.40%), *Vibrio* (1.24%), and so on (Fig 2A). Marine medaka gut microbiota was composed of 8 Phylum, 33 Class, 34 Order, 147 Family, 250 Genera, and 36 Species from a total of 4 samples. Among which, the main genera included the *Ruegeria* (28.32%), *Vibrio* (9.10%), *PeM15* (5.64%), *Haloferula* (4.54%), *Shewanella* (1.67%), and so on (Fig 2B). The freshwater medaka gut microbiota was composed of 4 Phylum, 39 Class, 29 Order, 140 Family, 149 Genera, and 66 Species from a total of 4 samples. And, the main genera were the *ZOR0006* (22.20%), *Mycobacterium* (14.56%), *Aeromonas* (12.55%), *Cetobacterium* (9.26%), *Pseudomonas* (7.65%), *Bacillus* (3.39%), and so on (Fig 2C).

Compared to the natural community, based on the number of isolated bacteria, it was found that the culturable gut microbiota was composed of 14 genera in zebrafish, 12 genera in marine medaka, and 17 genera in freshwater medaka (Fig 2D-F). Among the culturable bacteria in zebrafish, the dominate genera were the *Pseudomonas* (20.64%), *Acinetobacter* (12.89%), *Aeromonas* (10.27%), *Plesiomonas* (10.27%), *Shewanella* (7.65%), *Vibrio* (12.89%), *Bacillus* (5.14%), *etc* (Fig 2D). In marine medaka, the culturable genera involved mainly the *Vibrio* (43.56%), *Bacillus* (10.89%), *Pseudomonas* (6.93%), *Dietzia* (10.34%), *Rhodococcus* (3.96%), and *Acinetobacter* (3.96%) (Fig 2E). From the freshwater medaka, the culturable genera composed of the *Aeromonas* (13.40%), *Shewanella* (10.31%), *Bacillus* (10.31%), *Microbacterium* (10.31%), *Pseudomonas* (6.19%), *Vibrio* (6.19%), *Acinetobacter* (6.19%) and so on (Fig 2F).

To evaluate the representativeness of the isolate collection, we compared the culturable genera with the genus-level profiles identified by 16S rRNA gene sequencing of the original gut microbiota. The clear differences between the natural microbiota and the cultured bacterial collections in all three fish species were discovered. In zebrafish, sequencing data were dominated by Cetobacterium (33.02%) and Aeromonas (10.34%), whereas the isolated community was enriched in Pseudomonas (20.64%), Acinetobacter (12.89%), Vibrio (12.89%), and Aeromonas (10.27%). In marine medaka, Ruegeria (28.32%) and Xanthomarina (18.95%) were the dominant genera, while Vibrio (43.56%) became the predominant genus in the cultured collection. In freshwater medaka, sequencing showed relatively high abundances of ZOR0006 (22.20%), Mycobacterium (14.56%), Aeromonas (12.55%), and Cetobacterium (9.26%), whereas the cultured isolates were mainly represented by Aeromonas (13.40%), Shewanella (10.31%), Bacillus (10.31%), and Microbacterium (10.31%). Overall, the culture-based approach recovered only part of the natural gut microbiota and preferentially enriched several genera that were either low in abundance or not dominant in the sequencing data, indicating the presence of cultivation bias.

### 3.2. Isolated and identified bacterial strains from adult zebrafish intestines

**Fig 3:**
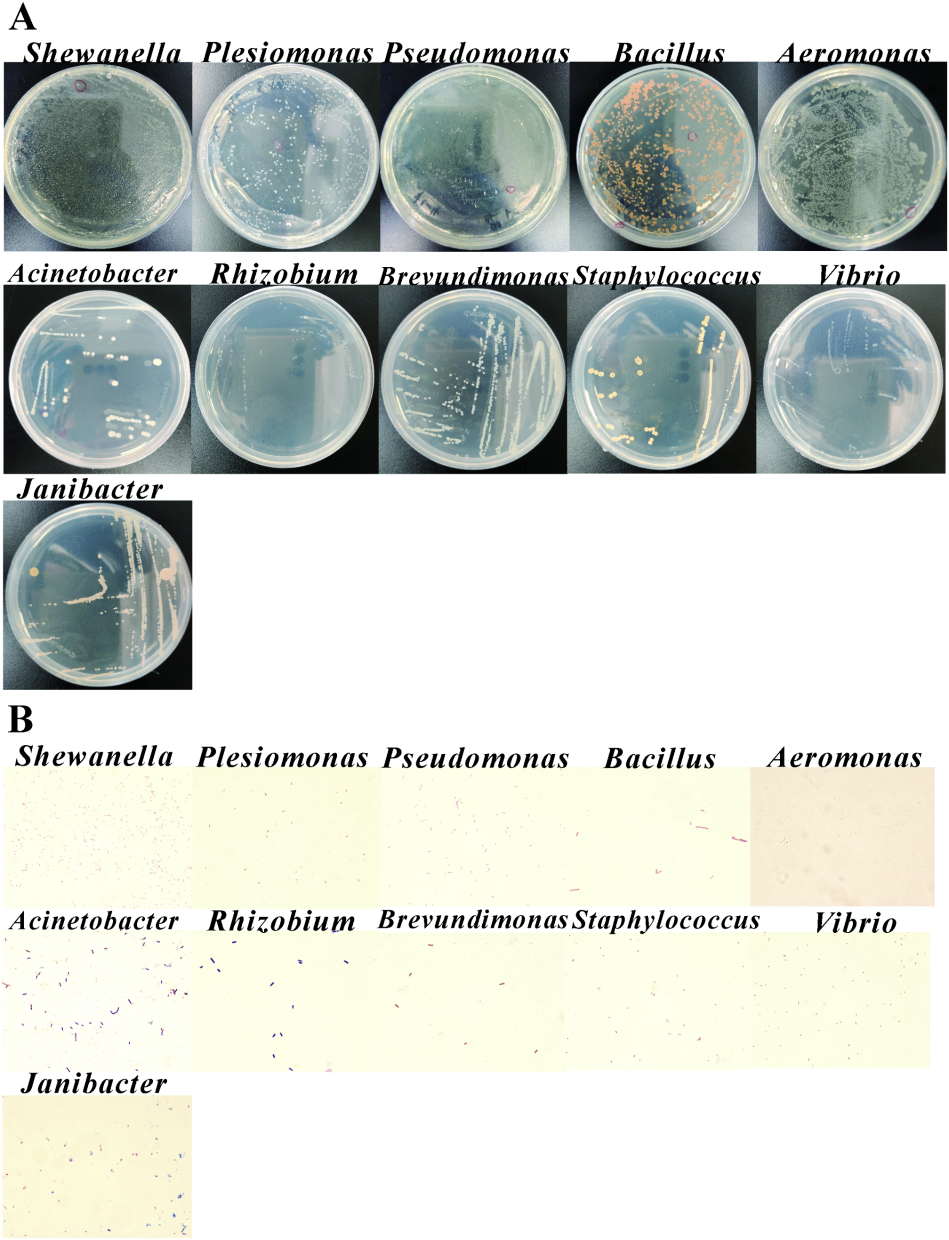
The characteristics of isolated intestinal bacteria from the adult zebrafish. **(A)** It represented the bacterial colonies on plates with individual strains purified and genus identified. **(B)** The detection of representative strains with gram stain.

A total of 78 bacterial isolates belonging to 14 genera were recovered from the gut microbiota of adult zebrafish (Table 1). These isolates were obtained under different sampling and cultivation conditions, including intestinal dissection or live-fish sampling, combined with aerobic or anaerobic culture (Fig 3). Overall, the zebrafish isolate collection showed clear genus-level diversity, and the composition of recovered bacteria varied among the different isolation conditions.

**Table 1:** The isolated and identified bacterial strains from adult zebrafish intestines under aerobic and anaerobic conditions.

Dissected intestines-Aerobic conditions-16 strains-8 genera							
**No.**	**Characters of colony**	**Blast matched results-16S database**	**Identity(%)**	**Genus group**	**Sequence length (bp)**	**Access No.**	**The Gram-detections**
1	Milk white color, small size and round, half-transparent.	Pseudomonas pseudoalcaligenes strain CH 1-1-1 (KM871859.1)	>99.9	*Pseudomonas*	1409	MK178498	Negative (G-)
2	Light yellow color, middle size and round, non-transparent.	Aeromonas veronii strain MJ-4 (KC210767.1)	100	*Aeromonas*	1411	MK178499	Negative (G-)
3	Brown red color, middle size and round, non-transparent.	Shewanella sp. lam-5 (KR072679.1)	>99.5	*Shewanella*	1422	MK178500	Negative (G-)
4	Light yellow color, small size and round, half-transparent.	Vibrio cholerae strain DL3 (MG062859.1)	>99.6	*Vibrio*	1411	MK178501	Negative (G-)
5	Yellow color, middle size and round, non-transparent.	Microbacterium sp. 0702P1-2 (HM222654.1)	100	*Microbacterium*	1404	MK178502	Negative (G-)
6	Milk white color, middle size and round, non-transparent.	Rhodococcus erythropolis strain HX-2 (MG015900.1)	100	*Rhodococcus*	1393	MK178503	Negative (G-)
7	Red color, middle size and round, non-transparent.	Exiguobacterium sp. strain Firmi-40 (MH683129.1)	>99.9	*Exiguobacterium*	1444	MK178504	Positive (G+)
8	Milk white color, middle size and round, half-transparent.	Bacillus sp. (in: Bacteria) strain JSM 1685003 (MG893110.1)	>99.9	*Bacillus*	1430	MK178505	Positive (G+)
**Dissected intestines-Anaerobic conditions-15 strains-7 genera**							
**No.**	**Characters of colony**	**Blast matched results-16S database**	**Identity(%)**	**Genus group**	**Sequence length (bp)**	**Samples’ name and Similarity**	**The Gram-detections**
1	Yellow color, big size and round, half-transparent.	Shewanella oneidensis strain MR-1	98.63%	*Shewanella*	1651	No.1 = ZF-Y-1;	Negative (G-)
2	Milk white color, small size and round, half-transparent.	Vibrio paracholerae strain EDC-792	95.85% (probably new species)	*Vibrio*	1445	No.2 = ZF-Y-2; No.6 = ZF-Y-6 = 96.08%; No.8 = ZF-Y-8 = 98.33%; No.10 = ZF-Y-10 = 98.40%; No.12 = ZF-Y-12 = 96.08%; No.15 = ZF-Y-15 = 95.86%; No.2 = No.6 = No.12 = 99.86%; No.2 = No.15 = 99.79%; No.6 = No.12 = 100%;	Negative (G-)
3	Milk white color, middle size and round, non-transparent.	Plesiomonas shigelloides strain NCIMB 9242	99.65%	*Plesiomonas*	1441	No.3 = ZF-Y-3; No.7 = ZF-Y-7 = 99.51%; No.11 = ZF-Y-11 = 99.72%; No.14 = ZF-Y-14 = 99.51%;	Negative (G-)
4	Milk white color, small size and round, half-transparent.	Pseudomonas chengduensis strain MBR	98.84%	*Pseudomonas*	1472	No.4 = ZF-Y-4;	Negative (G-)
5	Milk white color, small size and round, half-transparent.	Pseudomonas sediminis strain PI11	99.78%	*Pseudomonas*	1439	No.5 = ZF-Y-5;	Negative (G-)
9	Orange red color, big size and round, half-transparent.	Bacillus infantis strain SMC 4352−1	99.93%	*Bacillus*	1451	No.9 = ZF-Y-9;	Negative (G-)
13	Milk white color, small size and round, half-transparent.	Aeromonas jandaei strain CDC0787−80	99.38%	*Aeromonas*	1447	No.13 = ZF-Y-13;	Negative (G-)
**Live fish-Aerobic conditions-27 strains-10 genera**							
**No.**	**Characters of colony**	**Blast matched results-16S database**	**Identity(%)**	**Genus group**	**Sequence length (bp)**	**Samples’ name and Similarity**	**The Gram-detections**
1	Yellow color, small size and round, half-transparent.	Acinetobacter bereziniae strain ATCC 17924	99.45%	*Acinetobacter*	1444	No.1 = ZF-X-1; No.13 = ZF-X-13 = 99.72%; No.16 = ZF-X-16 = 99.65%;	Positive (G+)
2	Yellow color, small size and round, half-transparent.	Vibrio paracholerae strain EDC-792	98.20%	*Vibrio*	1450	No.2 = ZF-X-2; No.4 = ZF-X-4 = 98.48%; No.6 = ZF-X-6 = 98.34%; No.9 = ZF-X-9 = 98.33%; No.10 = ZF-X-10 = 98.47%; No.11 = ZF-X-11 = 98.33%; No.12 = ZF-X-12 = 98.34%; No.22 = ZF-H-6 = 98.20%; No.24 = ZF-H-8 = 98.33%;	
3	Milk white, big size and round, half-transparent.	Acinetobacter johnsonii strain ATCC 17909	99.30%	*Acinetobacter*	1448	No.3 = ZF-X-3; No.7 = ZF-X-7 = 99.17%; No.8 = ZF-X-8 = 99.30%; No.14 = ZF-X-14 = 99.44%; No.17 = ZF-H-1 = 99.03%; No.18 = ZF-H-2 = 99.23%;	Positive (G+)
5	Yellow color, small size and round, half-transparent.	Pseudomonas putida strain NBRC 14164	98.89%	*Pseudomonas*	1445	No.5 = ZF-X-5;	
15	Green color, middle size and round, half-transparent.	Acinetobacter bereziniae strain XH901	99.79%	*Acinetobacter*	1445	No.15 = ZF-X-15;	Positive (G+)
19	Chartreuse color, middle size and round, half-transparent.	Pseudomonas peli strain R-20805	98.81%	*Pseudomonas*	1444	No.19 = ZF-H-3; No.21 = ZF-H-5 = 98.68%;	
20	Milk white, small size and round, half-transparent.	Plesiomonas shigelloides strain NCIMB 9242	99.44%	*Plesiomonas*	1446	No.20 = ZF-H-4;	Positive (G+)
25	Milk white, middle size and round, non-transparent.	Aeromonas veronii bv. veronii strain ATCC 35624	99.65%	*Aeromonas*	1451	No.25 = ZF-H-9;	
23	Light red, middle size and round, half-transparent.	Shewanella oneidensis strain MR-1	98.29%	*Shewanella*	783	No.23 = ZF-H-7;	
**Live fish-Anaerobic conditions-10 strains-10 genera**							
**No.**	**Characters of colony**	**Blast matched results-16S database**	**Identity(%)**	**Genus group**	**Sequence length (bp)**	**Samples’ name and Similarity**	**The Gram-detections**
1	Yellow color, middle size and round, half-transparent.	Pseudomonas sediminis strain PI11	99.86%	*Pseudomonas*	1444	No.1 = ZF-Y-1-live;	
2	Milk white color, small size and round, half-transparent.	Rhizobium lemnae strain L6-16	97.59%	*Rhizobium*	1463	No.2 = ZF-Y-2-live;	Positive (G+)
3	Milk white color, middle size and round, non-transparent.	Acinetobacter johnsonii strain ATCC 17909	99.58%	*Acinetobacter*	1445	No.3 = ZF-Y-3-live;	Negative (G-)
4	Milk white color, middle size and round, half-transparent.	Brevundimonas lenta strain DS-18	98.68%	*Brevundimonas*	1363	No.4 = ZF-Y-4-live;	Negative (G-)
5	Orange yellow color, middle size and round, non-transparent.	Staphylococcus saprophyticus subsp. saprophyticus ATCC 15305 = NCTC 7292	99.72%	*Staphylococcus*	1455	No.5 = ZF-Y-5-live;	Negative (G-)
6	Yellow color, middle size and round, non-transparent.	Pseudomonas putida strain NBRC 14164	98.96%	*Pseudomonas*	1445	No.6 = ZF-Y-6-live;	
7	Yellow color, big size and round, half-transparent.	Aeromonas veronii bv. veronii strain ATCC 35624	99.65%	*Aeromonas*	1449	No.7 = ZF-Y-7-live;	
8	Milk white color, small size and round, half-transparent.	Acinetobacter bereziniae strain ATCC 17924	99.72%	*Acinetobacter*	1443	No.8 = ZF-Y-8-live;	
9	Yellow color, small size and round, half-transparent.	Vibrio paracholerae strain EDC-792	98.33%	*Vibrio*	1444	No.9 = ZF-Y-9-live;	
10	Yellow color, small size and round, non-transparent.	Plesiomonas shigelloides strain NCIMB 9242	99.31%	*Plesiomonas*	1443	No.10 = ZF-Y-10-live;	
**Second selection-Anaerobic conditions-6 strains-5 genera**							
**No.**	**Characters of colony**	**Blast matched results-16S database**	**Identity(%)**	**Genus group**	**Sequence length (bp)**	**Samples’ name and Similarity**	**The Gram-detections**
1	Milk white color, small size and round, half-transparent.	Vibrio paracholerae strain EDC-792	95.86%	*Vibrio*	1445	No.2 = ZF-y-2; ZF-y-2 = ZF-Y-6 = ZF-Y-12==ZF-Y-15 = 99.58%; ZF-y-2 = ZF-Y-2 = 99.65%;	Negative (G-)
2	Mile white color, big size and round, half-transparent.	Plesiomonas shigelloides strain NCIMB 9242	99.65%	*Plesiomonas*	1438	No.3 = ZF-y-3;	Negative (G-)
3	Light green, small size and round, half-transparent.	Pseudomonas sediminis strain PI11	99.79%	*Pseudomonas*	1433	No.4 = ZF-y-4;	
4	Yellow color, small size and round, non-transparent.	Staphylococcus warneri strain AW 25	99.52%	*Staphylococcus*	1451	No.5 = ZF-y-5;	Positive (G+)
5	Orange yellow color, small size and round, non-transparent.	Janibacter anophelis strain H2.16B	99.58%	*Janibacter*	1424	No.6 = ZF-y-6;	Negative (G-)

Note: 1.The first 8 genus from the Dissected intestines-Aerobic conditions were reported in our previous study [Jia, 2019]. 2.It also presented the possible similar bacterial strains with the compared percentages. 3.The green mark genus stands for the new bacterial strain with the low (<96%) similar to species from database blast. 4.The different strains were selected as the typical strain from each genus for following gram and metabolic assay.

From dissected intestinal samples cultured under aerobic conditions, 16 isolates representing 8 genera were obtained. Representative genera included Shewanella, Vibrio, Plesiomonas, Pseudomonas, Bacillus, and Aeromonas. In details, the colony of the No.1 sample was belonged to the *Shewanella* genus, and the best-matched bacteria was *Shewanella oneidensis strain MR-1* with 98.63% identity and 1651 bp length (Table 1). The colony of the No.2 sample was belonged to the *Vibrio* genus, and the best-matched bacteria was *Vibrio paracholerae* strain EDC-792, with 95.85% identity and 1445 bp length. Similarly, samples No. 6, 8, 10, 12, and 15 showed sequence identities ranging from 95.86% to 98.40%. The colony of No.3 sample belonged to *Plesiomonas* genus, and the *Plesiomonas shigelloides* strain NCIMB 9242 was the best-matched bacteria. Samples No. 7, 11, and 14 exhibited sequence identities exceeding 99.51%. The colony of No.4 and 5 sample showed similarity to the *Pseudomonas* genus, the best-matched bacteria was *Pseudomonas chengduensis* strain MBR and *Pseudomonas sediminis* strain PI11, respectively. The colony of No.9 sample was blasted to be *Bacillus* genus, and the best-matched bacteria was *Bacillus infantis* strain SMC 4352−1 isolated from the blood of a newborn child with sepsis. The colony of No.13 sample was the *Aeromonas* genus, and the best-matched bacteria was *Aeromonas jandaei* strain CDC0787−80 isolated from clinical specimens. The characteristics of bacterial colonies are normally milk white or yellow color, middle size and round, half-transparent (the plates are shown in the Fig 3A**,** and gram stain in Fig 3B).

However, the samples from the live fish intestines showed the different new genus compared to the traditional dissected tissues, which indicates the possible to discover the gut microbial community by using various efforts. There were 27 strains and 10 genera of zebrafish gut microbiota under living aerobic conditions (Table 1). Representative genera identified under this condition included Acinetobacter, Vibrio, Pseudomonas, Plesiomonas, Shewanella, and Aeromonas. In detail, the colony of the No.1 sample was belonged to the *Acinetobacter* genus, and the best-matched bacteria was *Acinetobacter bereziniae* strain ATCC 17924 isolated from human specimens. The colony of the No.2 was the *Vibrio* genus, the best-matched bacteria was *Vibrio paracholerae* strain EDC-792. BLAST analysis of the No. 4, 6, 9, 10, 11 samples and No.12 sample all showed sequence similarities greater than 98.32%. The colony of the No.3 was belonged to the *Acinetobacter* genus. The colony of the No.5 sample was belonged to the *Pseudomonas* genus, and the best-matched bacteria was *Pseudomonas putida* strain NBRC 14164. The NCBI comparison of the colony of the No.15 sample showed that the most compatible strain with it was *Bacterium strain* BS0230, but NCBI only mentioned that it belonged to the bacterial category and did not provide detailed classification. The second match for it was *Acinetobacter bereziniae* strain XH901, which belonged to the *Acinetobacter* genus, with 99.79% identity and 1445 bp length. The colony of the No.19 sample and the No.21 sample belonged to the *Pseudomonas* genus. Sample No. 20 was assigned to Plesiomonas and showed the highest similarity to Plesiomonas shigelloides strain NCIMB 9242. Sample No. 23 belonged to Shewanella and was closest to Shewanella oneidensis strain MR-1, whereas sample No. 25 showed the highest similarity to Aeromonas veronii strain ATCC 35624. These results indicate that live sampling under aerobic conditions recovered a relatively diverse bacterial assemblage from adult zebrafish intestines.

Under anaerobic conditions, bacterial recovery also differed according to sampling method. From live-fish intestinal samples under anaerobic culture, 10 genera were identified (Table 1), including Pseudomonas, Rhizobium, Acinetobacter, Brevundimonas, Staphylococcus, Aeromonas, Vibrio, and Plesiomonas. Representative isolates included Pseudomonas sediminis, Rhizobium lemnae, Acinetobacter johnsonii, Brevundimonas lenta, Staphylococcus saprophyticus, and Aeromonas veronii as the closest-matched reference strains in the NCBI database. Colony morphology was again mainly milky white or yellow, medium-sized, round, and half-transparent (Fig 3A). In addition to the isolates obtained directly during primary cultivation, a secondary isolation step after refrigerated storage of the original plates yielded 6 additional strains belonging to 5 genera, including Vibrio, Plesiomonas, Pseudomonas, Staphylococcus, and Janibacter (Table 1). These late-emerging colonies further expanded the diversity of the zebrafish isolate library.

### 3.3. Isolated and identified bacterial strains from adult marine medaka intestines

**Fig 4:**
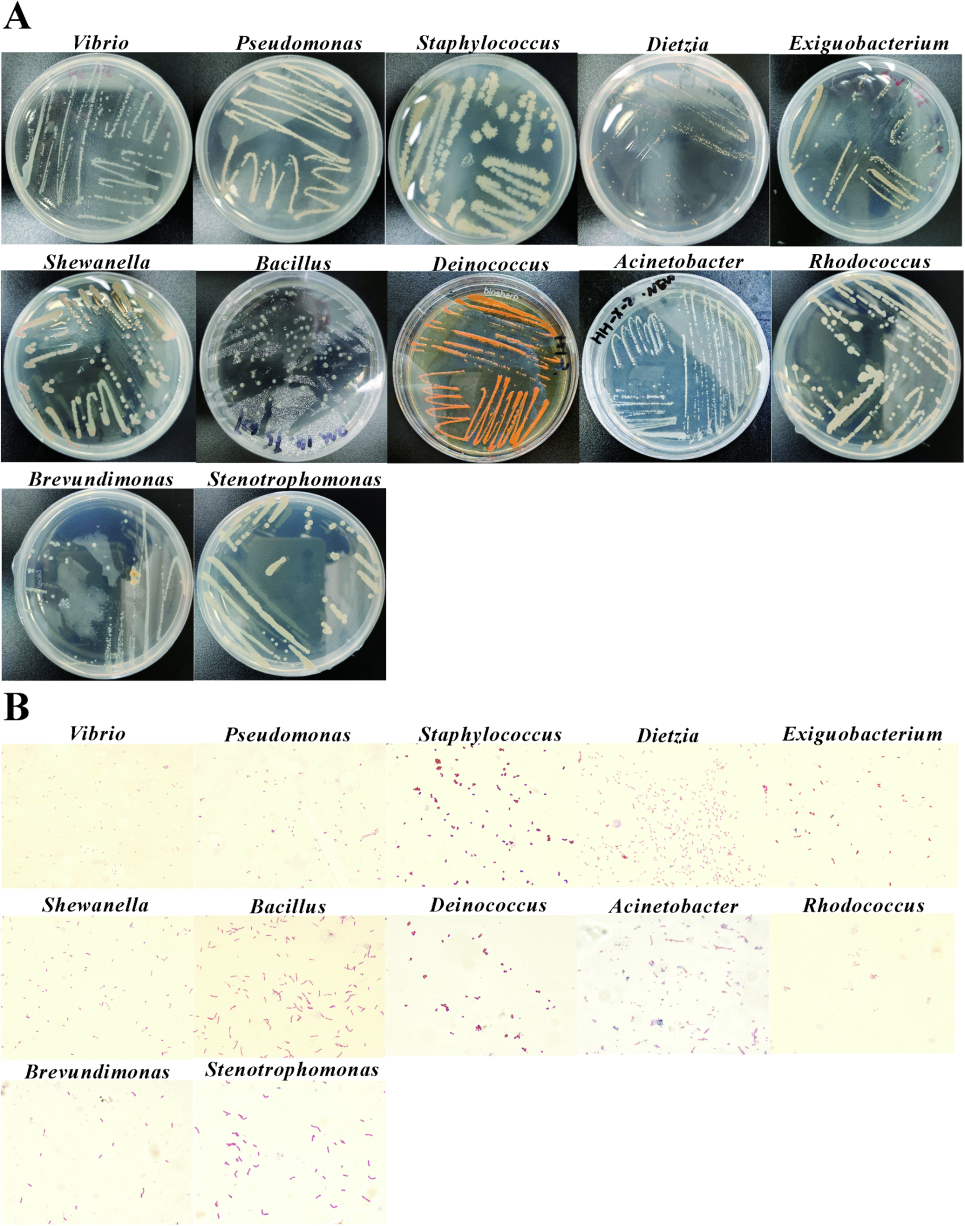
The characteristics of isolated intestinal bacteria from the adult marine medaka. **(A)** It represented the bacterial colonies on plates with individual strains purified and genus identified. **(B)** The detection of representative strains with gram stain.

A total of bacterial isolates from adult marine medaka were recovered under different sampling and cultivation conditions, including intestinal dissection or live-fish sampling combined with aerobic or anaerobic culture (Table 2). Overall, the recovered isolates represented 12 genera, and their composition varied depending on the sampling method and oxygen condition (Fig 4).

**Table 2:** The isolated and identified bacterial strains from adult marine medaka intestines under aerobic and anaerobic conditions.

Dissected intestines-Aerobic conditions-24 strains-9 genus							
**No.**	**Characters of colony**	**Blast matched results-16S database**	**Identity(%)**	**Genus group**	**Sequence length (bp)**	**Samples’ name and Similarity**	**The Gram-detections**
1.	Milk white color, middle size and round.	Vibrio alfacsensis strain CAIM 1831	99.59%	*Vibrio*	1460	No.1 = MM-1; No.3 = MM-3 = 99.45%; No.5 = MM-5 = 99.59%; No.7 = MM-7 = 99.45%; No.1-No.3-No.5 = 99.86%; No.1-No.3-No.5-No.7 = 99.79%;	Negative (G-)
2.	Yellow color, small size and round.	Vibrio paracholerae strain EDC-792	98.26%	*Vibrio*	1447	No.2 = MM-2 No.4 = MM-4 = 98.13%; No.2-No.4 = 100%;	Negative (G-)
6	Middle size and round, half-transparent.	Vibrio panuliri strain LBS2	99.79%	*Vibrio*	1457	No.6 = MM-6;	Negative (G-)
8	Light yellow color, middle size and round.	Pseudomonas veronii strain CIP 104663	99.79%	*Pseudomonas*	1442	No.8 = MM-8; No.9 = MM-9 = 99.58%; No.12 = MM-12 = 99.65%; No.13 = MM-13 = 99.79%; No.8-No.9 = 99.72%; No.8-No.12 = 99.93%; No.9-No.12-No.13 = 99.86%; No.8-No.13 = 99.65%;	Negative (G-)
10	Light green color, big size and round.	Pseudomonas khazarica strain TBZ2	99.72%	*Pseudomonas*	1442	No.10 = MM-10; No.15 = MM-15 = 99.37%; No.16 = MM-16 = 99.58%; No.22 = MM-22 = 99.58%; No.10-No.15-No.16 = 99.79%; No.10-No.22 = 99.86%;	Negative (G-)
11	Light yellow color, big size and irregular shape.	Staphylococcus succinus subsp. succinus strain AMG-D1	99.59%	*Staphylococcus*	1461	No.11 = MM-11； No.14 = MM-14 = 99.52%； No.18 = MM-18 = 99.72%; No.21 = MM-21 = 99.58%; No.21 = MM-21 = 99.58%; No.24 = MM-24 = 99.72%; No.14-No.18 = 99.66%; No.14-No.21 = 99.79%;	Positive (G+)
19	Light orange color, small size and round, non-transparent.	Dietzia aurantiaca strain CCUG 35676	99.14%	*Dietzia*	1430	N0.19 = MM-19;	Negative (G-)
20	Orange yellow color, small size and round.	Exiguobacterium aestuarii strain TF-16	99.66%	*Exiguobacterium*	1467	No.20 = MM-20；	Negative (G-)
23	Orange yellow color, middle size and round.	Shewanella seohaensis strain S7-3	99.58%	*Shewanella*	1446	No.23 = MM-23；	Negative (G-)
**Dissected intestines-Anaerobic conditions-19 strains-7 genus**							
**No.**	**Characters of colony**	**Blast matched results-16S database**	**Identity(%)**	**Genus group**	**Sequence length (bp)**	**Samples’ name and Similarity**	**The Gram-detections**
1	Light yellow color, small size and round, transparent.	Vibrio brasiliensis LMG 20546	98.47%	*Vibrio*	1454	No.1 = OM-Y-1; No.3 = OM-Y-3 = 98.27%; No.4 = OM-Y-4 = 98.14%; No.5 = OM-Y-5 = 98.27%; No.7 = OM-Y-7 = 98.46%; No.16 = OM-Y-16 = 98.14%; No.17 = OM-Y-17 = 98.27%; No.18 = OM-Y-18 = 98.27%;	Negative (G-)
2	Light yellow color, small size and round, transparent.	Vibrio vulnificus NBRC 15645 = ATCC 27562	98.75%	*Vibrio*	1445	No.2 = OM-Y-2; No.8 = OM-Y-8 = 98.82%;	Negative (G-)
6	Light yellow color, small size and round, transparent.	Vibrio owensii CAIM 1854 = LMG 25443 strain DY05	99.72%	*Vibrio*	1451	No.6 = OM-Y-6;	Negative (G-)
9	Milk white color, big size and round, transparent.	Vibrio fluvialis strain NBRC 103150	99.65%	*Vibrio*	1452	No.9 = OM-Y-9; No.10 = OM-Y-10 = 99.65%; No.19 = OM-Y-19 = 99.65%; No.9-No.10 = 99.79%;	Negative (G-)
11	Milk white color, big size and round, transparent.	Vibrio sp. strain PrVb096	99.86%	*Vibrio*	1453	No.11 = OM-Y-11; No.12 = OM-Y-12 = 99.72%;	Negative (G-)
13	Milk white color, big size and round, transparent.	Vibrio sp. strain PrVr101	100%	*Vibrio*	1453	No.13 = OM-Y-13;	Negative (G-)
14	Milk white color, big size and round, half-transparent.	Mesobacillus thioparans strain BMP-1	99.17%	*Bacillus*	1456	No.14 = OM-Y-14; No.15 = OM-Y-15 = 99.24%;	Negative (G-)
**Live fish-Aerobic conditions-15 strains-7 genus**							
**No.**	**Characters of colony**	**Blast matched results-16S database**	**Identity(%)**	**Genus group**	**Sequence length (bp)**	**Samples’ name and Similarity**	**The Gram-detections**
1	Milk white color, big size and round.	Bacillus cereus strain CCM 2010	99.79%	*Bacillus*	1454	No.1 = HH-1 No.2 = HH-2； No.4 = HH-4； No.7 = HH-7； No.8 = HH-8； No.14 = HH-X-7；	Positive (G+)
3	Orange color, small size and round.	Deinococcus proteolyticus strain MRP	99.57%	*Deinococcus*	1405	No.3 = HH-3;	Negative (G-)
5	Yellow color, big size and round.	Bacillus sanguinis strain BML-BC004	99.65%	*Bacillus*	1461	No.5 = HH-5； No.6 = HH-6； No.9 = HH-9； No.10 = HH-X-1	
11	White color, middle size and round.	Acinetobacter venetianus RAG-1 = CIP 110063 strain ATCC 31012	99.72%	*Acinetobacter*	1439	No.11 = HH-X-2;	Negative (G-)
12	Half-transparent, small size and round.	Vibrio owensii CAIM 1854 = LMG 25443 strain DY05	99.58%	*Vibrio*	1454	No.12 = HH-X-3； No.16 = HH-X-9;	
13	Light yellow color, small size and round.	Vibrio alfacsensis strain CAIM 1831	99.59%	*Vibrio*	1454	No.13 = HH-X-5；	
**Live fish-Anaerobic conditions-0 strains-0 genus**							
**Second selection-Anaerobic conditions-5 strains-5 genus**							
**No.**	**Characters of colony**	**Blast matched results-16S database**	**Identity(%)**	**Genus group**	**Sequence length (bp)**	**Samples’ name and Similarity**	**The Gram-detections**
1	Light yellow color, middle size and round, non-transparent.	Rhodococcus qingshengii strain CCM 4446	99.58%	*Rhodococcus*	1418	No.1 = OM-y-1;	Negative (G-)
2	Milk white color, middle size and round, half-transparent.	Brevundimonas bullata strain NBRC 13290	99.63%	*Brevundimonas*	1363	No.2 = OM-y-2;	
3	Light yellow color, middle size and round, non-transparent.	Stenotrophomonas bentonitica strain BII-R7	99.58%	*Stenotrophomonas*	1445	No.3 = OM-y-3;	Negative (G-)
4	Milk white color, small size and round, half-transparent.	Staphylococcus saprophyticus subsp. saprophyticus ATCC 15305 = NCTC 7292	99.65%	*Staphylococcu*	1453	No.4 = OM-y-4;	Negative (G-)
5	Milk white color, big size and irregular shape transparent.	Vibrio fluvialis strain NBRC 103150	99.86%	*Vibrio*	1448	No.5 = OM-y-5;	

Note: 1.It presented the possible similar bacterial strains with the compared percentages. 2.The different strains were chosen as the typical strain from each genus for following gram and metabolic assay.

From dissected intestinal samples cultured under aerobic conditions, 24 isolates belonging to 9 genera were obtained. In details, the colony of the No.1 sample was *Vibrio* genus, and the best-matched bacteria was *Vibrio alfacsensis sp*. nov., isolated from marine organisms [33](Table 2). The colony of the No.2 and the No.4 sample was belonged to the *Vibrio* genus, with the best-matched species being *Vibrio paracholerae strain* EDC-792, with 98.26% identity and a length of 1447 bp (Table 2). The colony of the No.6 sample possibly belonged to the genus *Vibrio*, with the best-matched bacteria of *Vibrio owensii sp.* nov., isolated from spiny lobster from Andaman sea [34]. The colony of the No.8 sample belonged to the *Pseudomonas* genus, the best-matched bacteria was *Pseudomonas veronii* strain CIP 104663, isolated from natural mineral water, with 99.79% identity and 1442 bp length (Table 2). The No.9, 12, and 13 sample were similar. The colony of the No.10 sample was belonged to the *Pseudomonas* genus, with the best-matched bacteria being *Pseudomonas khazarica sp.* nov., a polycyclic aromatic hydrocarbon-degrading bacterium isolated from Khazar Sea sediments [35]. The colony of the No.11 sample seemed to belong to S*taphylococcus* genus with the best-matched bacteria *Staphylococcus succinus* sp. nov., isolated from Dominican amber, with 99.59% identity and 1461 bp length (Table 2). As for the No.19, it was belonged to the genus *Dietzia*, with the best-matched bacteria *Dietzia aurantiaca sp.* nov., isolated from a human clinical specimen, with 99.14% identity and a length of 1430 bp (Table 2). The No.20 sample was matched with *Exiguobacterium aestuarii* strain TF-16 from a tidal flat of the Yellow-sea in Korea (Table 2). The No.23 sample was from *Shewanella* genus, with the best-matched species being *Shewanella seohaensis* sp. Nov., isolated from tidal flat sediment (Table 2). These results indicate that aerobic cultivation of dissected intestinal samples recovered a relatively diverse bacterial assemblage from adult marine medaka.

Under anaerobic conditions of intestinal dissection, 19 strains and 7 genera of intestinal microbiota were obtained from marine medaka. In contrast to the aerobic condition, the isolate composition was strongly dominated by the genus Vibrio, with multiple samples showing high similarity to different Vibrio reference strains, including Vibrio brasiliensis, Vibrio vulnificus, Vibrio owensii, Vibrio fluvialis, and Vibrio sp. Additional isolates belonged to Bacillus-related taxa, including Mesobacillus thioparans as the closest match. These findings suggest that anaerobic cultivation of dissected samples enriched a narrower but still distinct subset of the marine medaka gut microbiota.

Under living aerobic conditions, 15 strains and 7 genera of marine medaka intestinal microbiota were obtained. In details, the colony of the No.1 sample was belonged to the *Bacillus* genus, with the best-matched bacteria of *Bacillus cereus* strain CCM 2010 isolated from air in the cowshed. BLAST analysis of the No.2, 4, 7, 8, and 14 samples showed that they were the same with 100% identity (Table 2). The colony of the No.3 sample belonged to the *Deinococcus* genus, with the best-matched bacteria of *Deinococcus proteolyticus* strain MRP, isolated from Lama glama’s faces. The colony of No.5, 6 and 9 samples belonged to the *Bacillus* genus, with the best-matched bacteria of *Bacillus sanguinis* strain BML-BC004. The colony of No.11 sample belonged to the *Acinetobacter* genus, with the best-matched bacteria of *Acinetobacter* *venetianus* RAG-1, a highly efficient oil-degrading bacterium isolated from the crude oil in Venice Bay pollutes the seawater (Table 2). The colony of No.12 sample belonged to the *Vibrio* genus, with the best-matched bacteria of *Vibrio owensii* CAIM 1854. While, the colony of No.13 sample also was identified as the *Vibrio* genus, and the best-matched bacteria *Vibrio alfacsensis* strain CAIM 1831 was isolated from marine organisms (Table 2). Under the living anaerobic conditions, no strain was isolated based on the colony characteristics. Among the isolated genera, the major strains belonged to *Pseudomonas*, *Shewanella*, *Bacillus* and *Vibrio* in dominant phylum of Proteobacteria in marine medaka. Taken together, the culture-derived bacterial community of adult marine medaka was mainly composed of members of Vibrio, Pseudomonas, Shewanella, and Bacillus, with clear differences among sampling and cultivation conditions. In particular, Vibrio was the most prominent genus recovered from dissected samples, especially under anaerobic conditions, whereas live aerobic sampling yielded a smaller but still diverse set of isolates. These results demonstrate that both sampling strategy and oxygen condition influenced the bacterial genera recovered from adult marine medaka intestines.

### 3.4. Isolated and identified bacterial strains from freshwater medaka intestines

**Fig 5:**
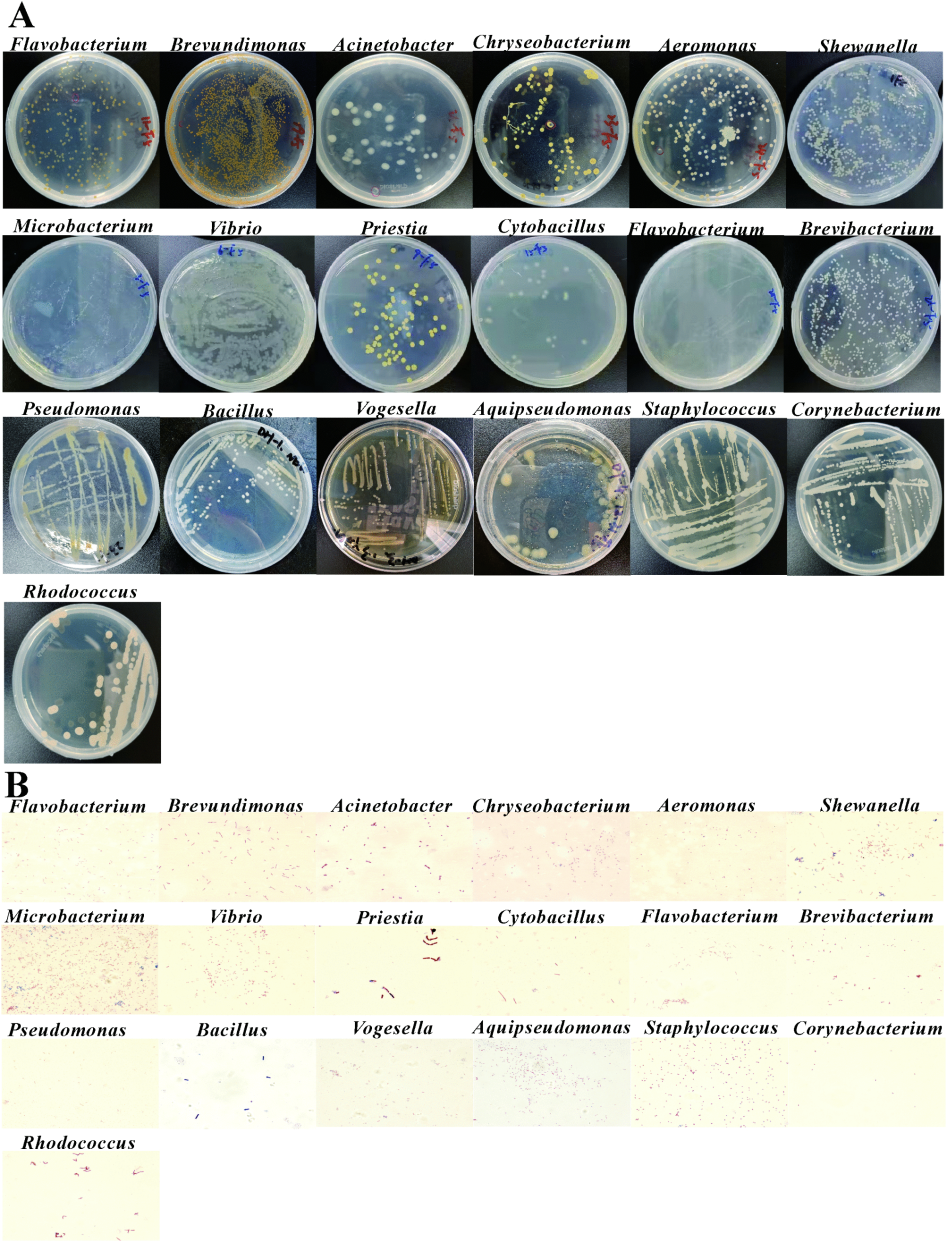
The characteristics of isolated intestinal bacteria from the adult freshwater medaka. **(A)** It represented the bacterial colonies on plates with individual strains purified and genus identified. **(B)** The detection of representative strains with gram stain.

A total of bacterial isolates from adult freshwater medaka were recovered under different sampling and cultivation conditions, including intestinal dissection or live-fish sampling combined with aerobic or anaerobic culture (Table 3). Overall, the culture-derived bacterial collection represented 18 genera, indicating a relatively broad genus-level diversity in the gut microbiota of adult freshwater medaka (Fig 5).

**Table 3:** The isolated and identified bacterial strains from adult freshwater medaka intestines under aerobic and anaerobic conditions.

Dissected intestines-Aerobic conditions-26 strains-9 genus							
**No.**	**Characters of colony**	**Blast matched results-16S database**	**Identity(%)**	**Genus group**	**Sequence length (bp)**	**Samples’ name and Similarity**	**The Gram-detections**
1	Milk white color, middle size and round.	Aeromonas veronii bv. veronii strain ATCC 35624	99.51%	*Aeromonas*	1445	No.1 = OL-H-1; No.4 = OL-H-4 = 99.58%; No.13 = OL-H-13 = 99.38%; No.22 = OL-H-22 = 99.65%; No.25 = OL-H-25 = 99.79%;	Negative (G-)
2	Milk white color, big size and round.	Vibrio fluvialis strain NBRC 103150	99.86%	*Vibrio*	1454	No.2 = OL-H-2; No.3 = OL-H-3 = 99.86%; No.5 = OL-H-5 = 99.66%; No.7 = OL-H-7 = 99.86%; No.8 = OL-H-8 = 99.65%; No.9 = OL-H-9 = 99.86%; No.10 = OL-H-10 = 99.65%; No.12 = OL-H-12 = 99.86%; No.16 = OL-H-16 = 99.86%; No.17 = OL-H-17 = 99.86%;	Negative (G-)
6	Milk white color, big size and round.	Shewanella oneidensis strain MR-1	98.57%	*Shewanella*	1481	No.6 = OL-H-6;	Positive (G+)
11	Light yellow color, middle size and round.	Flavobacterium succinicans strain NBRC 14905	99.57%	*Flavobacterium*	1413	No.11 = OL-H-11;	Negative (G-)
14	Light red color, big size and round.	Shewanella xiamenensis strain S4	98.69%	*Shewanella*	1427	No.14 = OL-H-14; No.15 = OL-H-15 = 98.50%; No.18 = OL-H-18 = 98.57%;	Negative (G-)
19	Orange yellow color, small size and round.	Brevundimonas staleyi strain FWC43	98.53%	*Brevundimonas*	1363	No.19 = OL-H-19;	Negative (G-)
20	Milk white color, big size and round.	Acinetobacter johnsonii strain ATCC 17909	99.02%	*Acinetobacter*	1439	No.20 = OL-H-20;	Negative (G-)
21	Milk white color, big size and round.	Acinetobacter lwoffii strain DSM 2403	99.44%	*Acinetobacter*	1440	No.21 = OL-H-21;	Negative (G-)
23	Yellow color, big size and round.	Chryseobacterium binzhouense strain lm2	99.71%	*Chryseobacterium*	1425	No.23 = OL-H-23; No.26 = OL-H-26 = 98.93%;	Negative (G-)
24	Light yellow color, big size and round.	Aeromonas jandaei strain CDC0787−80	99.86%	*Aeromonas*	1478	No.24 = OL-H-24;	Negative (G-)
**Dissected intestines-Anaerobic conditions-24 strains-12 genus**							
**No.**	**Characters of colony**	**Blast matched results-16S database**	**Identity(%)**	**Genus group**	**Sequence length (bp)**	**Samples’ name and Similarity**	**The Gram-detections**
1	Light red color, big size and round.	Shewanella oneidensis strain MR-1	98.62%	*Shewanella*	1478	No.1 = OL-Y-1; No.12 = OL-Y-12 = 98.30%; No.14 = OL-Y-14 = 98.57%;	Negative (G-)
2	Milk white color, big size and round.	Aeromonas veronii bv. veronii strain ATCC 35624	99.44%	*Aeromonas*	1445	No.2 = OL-Y-2; No.10 = OL-Y-10 = 99.51%; No.13 = OL-Y-13 = 99.45%; No.16 = OL-Y-16 = 99.79%; No.17 = OL-Y-17 = 99.52%;	Negative (G-)
3	Orange color, small size and round half-transparent.	Microbacterium zeae strain 1204	98.79%	*Microbacterium*	1427	No.3 = OL-Y-3; No.4 = OL-Y-4 = 99.07%; No.7 = OL-Y-7 = 99.00%; No.8 = OL-Y-8 = 98.93%; No.11 = OL-Y-11 = 99.00%; No.18 = OL-Y-18 = 99.00%;	Negative (G-)
5	Light green color, small size and round.	Microbacterium dextranolyticum strain DSM 8607	98.59%	*Microbacterium*	1425	No.5 = OL-Y-5;	Negative (G-)
6	Milk white color, big size and round, half-transparent.	Vibrio fluvialis strain NBRC 103150	99.65%	*Vibrio*	1451	No.6 = OL-Y-6;	Negative (G-)
9	Yellow color, big size and round.	Priestia aryabhattai B8W22	99.72%	*Priestia*	1459	No.9 = OL-Y-9;	Negative (G-)
15	Light yellow color, big size and round.	Cytobacillus firmus strain NBRC 15306	98.83%	*Cytobacillus*	1457	No.15 = OL-Y-15;	Negative (G-)
19	Yellow color, small size and round.	Microbacterium algeriense strain G1	99.65%	*Microbacterium*	1433	No.19 = OL-Y-19;	Negative (G-)
20	Light yellow color, small size and round.	Flavobacterium succinicans strain NBRC 14905	99.44%	*Flavobacterium*	1423	No.20 = OL-Y-20;	Negative (G-)
21	Milk white color, middle size and round.	Brevibacterium casei strain DSM 20657	98.93%	*Brevibacterium*	1433	No.21 = OL-Y-21;	Negative (G-)
22	Light yellow color, small size and round.	Pseudomonas alcaligenes strain ATCC 14909	98.62%	*Pseudomonas*	1478	No.22 = OL-Y-22;	Negative (G-)
23	Purple color, middle size and round.	Pseudomonas aeruginosa strain DSM 50071	99.72%	*Pseudomonas*	1439	No.23 = OL-Y-23; No.24 = OL-Y-24 = 99.65%;	Negative (G-)
**Live fish-Aerobic conditions-3 strains-3 genus**							
**No.**	**Characters of colony**	**Blast matched results-16S database**	**Identity(%)**	**Genus group**	**Sequence length (bp)**	**Samples’ name and Similarity**	**The Gram-detections**
1	Orange yellow color, big size and round.	Bacillus wiedmannii strain FSL W8-0169	99.65%	*Bacillus*	1465	No.1 = DH-1	Positive (G+)
2	Milk white color, middle size and round, half-transparent.	Bacillus cereus strain CCM 2010	99.72%	*Bacillus*	1457	No.2 = DH-2； DH-2 = DY-3 = 99.52%; DH-2 = DY-5 = 99.65%;	
3	Light yellow color, middle size and round, half-transparent.	Vogesella urethralis strain YM-1	99.71%	*Vogesella*	1449	No.3 = DH-3;	Negative (G-)
**Live fish-Anaerobic conditions-6 strains-3 genus**							
**No.**	**Characters of colony**	**Blast matched results-16S database**	**Identity(%)**	**Genus group**	**Sequence length (bp)**	**Samples’ name and Similarity**	**The Gram-detections**
1	Light yellow color, big size and round.	Aeromonas veronii bv. veronii strain ATCC 35624	99.72%	*Aeromonas*	1446	No.1 = DY-1;	Negative (G-)
2	Yellow color, small size and round.	Bacillus sanguinis strain BML-BC004	99.93% 2	*Bacillus*	1456	No.2 = DY-2； No.6 = DY-6； DY-2 = DY-6 = 99.52%	
3	Orange color, small size and round, wet.	Bacillus cereus strain CCM 2010	99.52%	*Bacillus*	1456	No.3 = DY-3； No.5 = DY-5； DY-3 = DY-5 = 99.93%;	
4	Milk white color, middle size and round.	Aquipseudomonas alcaligenes strain NBRC 14159	99.72% 3	*Aquipseudomonas*	1438	No.4 = DY-4;	Negative (G-)
**Second selection-Anaerobic conditions-3 strains-3 genus**							
**No.**	**Characters of colony**	**Blast matched results-16S database**	**Identity(%)**	**Genus group**	**Sequence length (bp)**	**Samples’ name and Similarity**	**The Gram-detections**
1	Light yellow color, small size and round, non-transparent.	Staphylococcus saprophyticus subsp. saprophyticus ATCC 15305 = NCTC 7292	99.65%	*Staphylococcus*	1450	No.1 = OL-y-1;	Positive (G+)
2	Milk white color, middle size and round, non-transparent.	Corynebacterium casei LMG S-19264	99.58%	*Corynebacterium*	1421	No.2 = OL-y-2;	Positive (G+)
3	Orange yellow color, big size and round, non-transparent.	Rhodococcus qingshengii strain CCM 4446	99.93%	*Rhodococcus*	1414	No.3 = OL-y-3; OL-y-3 = OM-y-1 = 99.79%;	Negative (G-)

Note: 1.It presented the possible similar bacterial strains with the compared percentages. 2.The different strains were chosen as the typical strain from each genus for following gram and metabolic assay.

In the present study, under aerobic conditions for intestinal dissection, a total of 26 bacterial colonies belonging to 9 genera with the identity >98% were indicated. In details, the colony of the No.1 sample was belonged to the *Aeromonas* genus, with the best-matched bacteria being *Aeromonas veronii* strain ATCC 35624, isolated from human saliva. The colony of the No.2 sample was from *Vibrio* genus, with the best-matched bacteria being *Vibrio fluvialis* strain NBRC 103150. Moreover, samples No. 3, 5, 7, 8, 9, 10, 12, 16, and 17 all showed sequence identities greater than 99.65% (Table 3). Samples No. 6 and 14 were assigned to the genus Shewanella, with the closest matches being Shewanella oneidensis strain MR-1 and Shewanella xiamenensis strain S4, respectively, while samples No. 15 and 18 also showed similarity to Shewanella with 98.50% and 98.57% identity (Table 3). The colony of the No.11 sample was belonged to the *Flavobacterium* genus, with the best-matched bacteria being *Flavobacterium succinicans* strain NBRC 14905 isolated from fresh water environment (Table 3). The No.19 sample was belonged to the *Brevundimonas* genus, with the best-matched bacteria being *Brevundimonas staleyi* strain FWC43 isolated from activated sludge (Table 3). The No.20 sample was belonged to the *Acinetobacter* genus, with the best-matched bacteria being *Acinetobacter johnsonii* strain ATCC 17909 isolated from fresh water environment (Table 3). While the colony of the No.21 sample also was belonged to the *Acinetobacter* genus, with the best-matched bacteria being *Acinetobacter lwoffii* strain DSM 2403 isolated from soil. The colony of the No.23 sample was belonged to the *Chryseobacterium* genus, with the best-matched bacteria being *Chryseobacterium binzhouense* strain lm2, isolated from activated sludge. The colony of the No.24 sample was belonged to the *Aeromonas* genus, with the best-matched bacteria being *Aeromonas jandaei* strain CDC0787−80 isolated from human feces (Table 3).

Under anaerobic conditions of intestinal dissection, 24 strains and 12 genera of freshwater medaka intestinal microbiota were obtained. To be specific, the colony of the No.1 sample was belonged to the *Shewanella* genus, with the best-matched bacteria *Shewanella oneidensis* strain MR-1 isolated from fresh water environment. The colony of the No.2 sample was belonged to the *Aeromonas* genus, with the best-matched bacteria *Aeromonas veronii* strain ATCC 35624. The colony of the No.3 sample possibly was belonged to the genus *Microbacterium*, with the best-matched bacteria of *Microbacterium zeae* strain 1204 isolated from corn stem tissue (surface sterilized) in Fangshan District, Beijing, China. While, the colony of the No.5 sample also was one strain of the *Microbacterium* genus, but the best-matched bacteria was *Microbacterium dextranolyticum* strain DSM 8607, isolated from soil (Table 3). The colony of the No.6 sample was belonged to *Vibrio* genus with the best-matched bacteria *Vibrio fluvialis* strain NBRC 103150. The colony of the No.9 sample seemed to belong to *Priestia* genus with the best-matched bacteria *Priestia aryabhattai* B8W22, isolated from the stratospheric atmosphere in India. As for the No.15, it was blasted to be best matched with *Cytobacillus firmus* strain NBRC 15306. The colony of the No.19 sample was blasted with the best-matched bacteria *Microbacterium algeriense* strain G1, isolated from Oilfield production waters. The colony of the No.20 sample was belonged to the genus *Flavobacterium*, with the best-matched bacteria of *Flavobacterium succinicans* strain NBRC 14905. The colony of the No.21 sample was best matched to *Brevibacterium casei* strain DSM 20657, with 98.93% identity and 1433 bp length by NCBI–BLAST (Table 3). Samples No. 22 and 23 both belonged to the genus Pseudomonas, showing the highest similarity to Pseudomonas alcaligenes strain ATCC 14909 and Pseudomonas aeruginosa strain DSM 50071, respectively (Table 3).

Under living aerobic conditions, there were 3 strains and 3 genera of intestinal microbiota were obtained from freshwater medaka. The colony of the No.1 and 2 sample was belonged to the *Bacillus* genus, with the best-matched bacteria *Bacillus wiedmannii* strain FSL W8-0169, and *Bacillus cereus* strain CCM 2010 (Table 3). The colony of the No.3 sample was from the *Vogesella* genus, with the best-matched bacteria *Vogesella urethralis* strain YM-1, isolated from human urine (Table 3). Although the total number of isolates was limited, live-fish aerobic sampling still yielded representative bacterial taxa from the freshwater medaka intestine.

Under living anaerobic condition, there were 6 strains and 3 genera of freshwater medaka intestinal microbiota were obtained. The colony of the No.1 sample was belonged to the *Aeromonas* genus, with the best-matched bacteria *Aeromonas veronii* strain ATCC 35624 (Table 3). While the colony of the No.2, 6, and No.3, 5 samples were belonged to the *Bacillus* genus, with the best-matched bacteria *Bacillus sanguinis* strain BML-BC004, and *Bacillus cereus* strain CCM 20104 (Table 3). The No.4 sample was one bacterium of the *Aquipseudomonas* genus, with the best-matched bacteria *Aquipseudomonas alcaligenes* strain NBRC 14159 (Table 3).

Taken together, the culture-derived bacterial community of adult freshwater medaka was composed mainly of Aeromonas, Vibrio, Shewanella, Microbacterium, Pseudomonas, and several other freshwater-associated genera, with marked differences among sampling and cultivation conditions. In particular, dissected intestinal samples, especially under anaerobic conditions, produced the highest genus-level diversity, whereas live-fish sampling yielded fewer isolates and a narrower taxonomic range. These results indicate that both sampling method and oxygen condition strongly influenced the recovery of gut bacteria from adult freshwater medaka.

### 3.5. Gram stain of selected representative strains of major genera of fish gut bacteria

In the subsequent experiments, the representative strains of different bacterial genera were selected for the gram staining assay (from the Table 1–3, Figs 3–5) Under the aerobic conditions, it can be found that from intestinal dissection of zebrafish, there were 6 genera with Gram-negative stained, which were *Pseudomonas*, *Aeromonas*, *Shewanella*, *Vibrio*, *Microbacterium*, and *Rhodococcus*, and 2 genera of *Exiguobacterium* and *Bacillus* showed Gram-positive stain (Fig 3B). In the intestinal bacteria of marine medaka, there were 5 genera with Gram-negative, which included *Vibrio*, *Pseudomonas*, *Dietzia*, *Exiguobacterium*, and *Shewanella*, and 1 strain of *Staphylococcus* genus was Gram-positive (Fig 4B). In the intestinal bacteria of freshwater medaka, totally 7 genera were Gram-negative, which included *Aeromonas*, *Vibrio*, *Flavobacteriu*, *Shewanella*, *Brevundimonas*, *Acinetobacter*, and *Chryseobacterium.* Moreover, there were 2 strains of genera *Shewanella* and *Acinetobacter* that showed Gram-positive stain (Fig 5B).

Under anaerobic conditions of intestinal dissection, in zebrafish gut bacteria, 1 strain of *Staphylococcu* genus was Gram-positive, and 7 genera with Gram-negative stain, which were *Vibrio*, *Plesiomonas*, *Pseudomonas*, *Bacillus*, *Aeromonas*, *Shewanella*, and *Janibacter* (Fig 3B)*.* In the intestinal bacteria of marine medaka, 5 genera were Gram-negative stained, which were *Vibrio*, *Bacillus*, *Rhodococcus*, *Stenotrophomonas*, and *Staphylococcu* (Fig 4B). In the intestinal bacteria of freshwater medaka, 10 genera were Gram-negative stained, which were *Shewanella*, *Aeromonas*, *Microbacterium*, *Vibrio*, *Priestia*, *Cytobacillus*, *Flavobacterium*, *Brevibacterium*, *Pseudomonas*, and *Rhodococcus*. And 2 genera with Gram-positive stain, which were *Staphylococcus*, and *Corynebacterium* (Fig 5B).

Under living aerobic conditions, among the selected intestinal bacteria of zebrafish, there were 2 genera of *Acinetobacter* and *Plesiomonas* detected with Gram-positive stain (Fig 3B)*.* In the intestinal bacteria of marine medaka, 1 genus of *Bacillus* showed the Gram-positive, and 2 genera of *Deinococcus* and *Acinetobacter* were Gram-negative stain (Fig 4B). In the intestinal bacteria of freshwater medaka, 1 strain of *Bacillus* genus was Gram-positive, while 1 strain of *Vogesella* genus was Gram-negative stained (Fig 5B).

Under living anaerobic conditions, in the intestinal bacteria of zebrafish, there were 3 genera with Gram-negative stain, which were *Acinetobacter*, *Brevundimonas*, and *Staphylococcus*. And 1 genus of *Rhizobium* was Gram-positive stain (Fig 3B). In the intestinal bacteria of marine medaka, there were no genera isolated from live fish under anaerobic conditions. In the intestinal bacteria of freshwater medaka, 2 genera of *Aeromonas* and *Aquipseudomona* were with Gram-negative stained, and no genus was Gram-positive (Fig 5B).

### 3.6. Composition and phylogeny tree of culturable bacteria in different fish models

In the gut microbiota of zebrafish, the culturable 10 genera including *Pseudomonas*, *Vibrio*, *Acinetobacter*, *Bacillus*, *Microbacterium*, *Shewanella*, *Plesiomonas*, *Aeromonas*, *Rhodococcus*, *Exiguobacterium* were isolated as aerobic bacteria*.* Among which, the *Pseudomonas* genus was the most numerous with 6 strains. Anaerobes bacteria consist totally 10 genera, which were *Pseudomonas*, *Rhizobium*, *Staphylococcus*, *Acinetobacter*, *Brevundimonas*, *Shewanella*, *Plesiomonas*, *Aeromonas*, *Bacillus*, and *Pseudorhizobium*. In the gut microbiota of marine medaka, there were a total of 11 genera of aerobic bacteria which are *Vibrio*, *Pseudomonas*, *Staphylococcus*, *Dietzia*, *Exiguobacterium*, *Shewanella*, *Bacillus*, *Deinococcus*, *Acinetobacter*, *Flavobacterium*, and *Chryseobacterium*. Anaerobes microbiota consists of 2 genera were *Vibrio* and *Bacillus*. In the gut microbiota of freshwater medaka, there are a total of 9 genera of aerobic bacteria as *Aeromonas*, *Vibrio*, *Shewanella*, *Acinetobacter*, *Flavobacterium*, *Chryseobacterium*, *Brevundimonas*, *Bacillus*, and *Vogesella*. Anaerobes microbiota included 11 genera, such as *Shewanella, Aeromonas*, *Microbacterium*, *Vibrio*, *Priestia*, *Cytobacillus*, *Brevibacterium*, *Aquipseudomonas*, *Bacillus*, *Flavobacterium*, and *Pseudomonas*.

Firstly, in terms of diversity at genus level among aerobic bacteria, zebrafish has the highest bacterial diversity with 10 genera, followed by freshwater medaka and marine medaka, both of which have 9 genera. In terms of anaerobic bacteria, freshwater medaka has the highest diversity with 12 bacterial genera, and then it is zebrafish with 10 genera, while marine medaka has relatively low diversity of bacterial genera in intestinal samples because anaerobic bacteria were not isolated from live fish.

Secondly, the dominant bacterial genera of the three types of fish also have both similarities and differences. *Vibrio* and *Pseudomonas* are both relatively common aerobic bacteria in the three types of fish, but the advantage of the genus *Vibrio* is more obvious in marine medaka. The genus *Acinetobacter* was found in aerobic bacteria of both zebrafish and freshwater medaka, but not in marine medaka. Among the anaerobic bacteria in freshwater medaka, the genera *Shewanella* and *Microbacterium* are more prominent, while these two genera are found less or not at all in the anaerobic bacteria of zebrafish and marine medaka.

Finally, the genera *Staphylococcus* and *Dietzia* are unique to the aerobic bacteria in marine medaka, which may be related to its marine living environment. The anaerobic bacteria species in freshwater medaka are relatively rich, which may reflect the differences in microbial composition between the freshwater environment and the marine environment.

**Fig 6:**
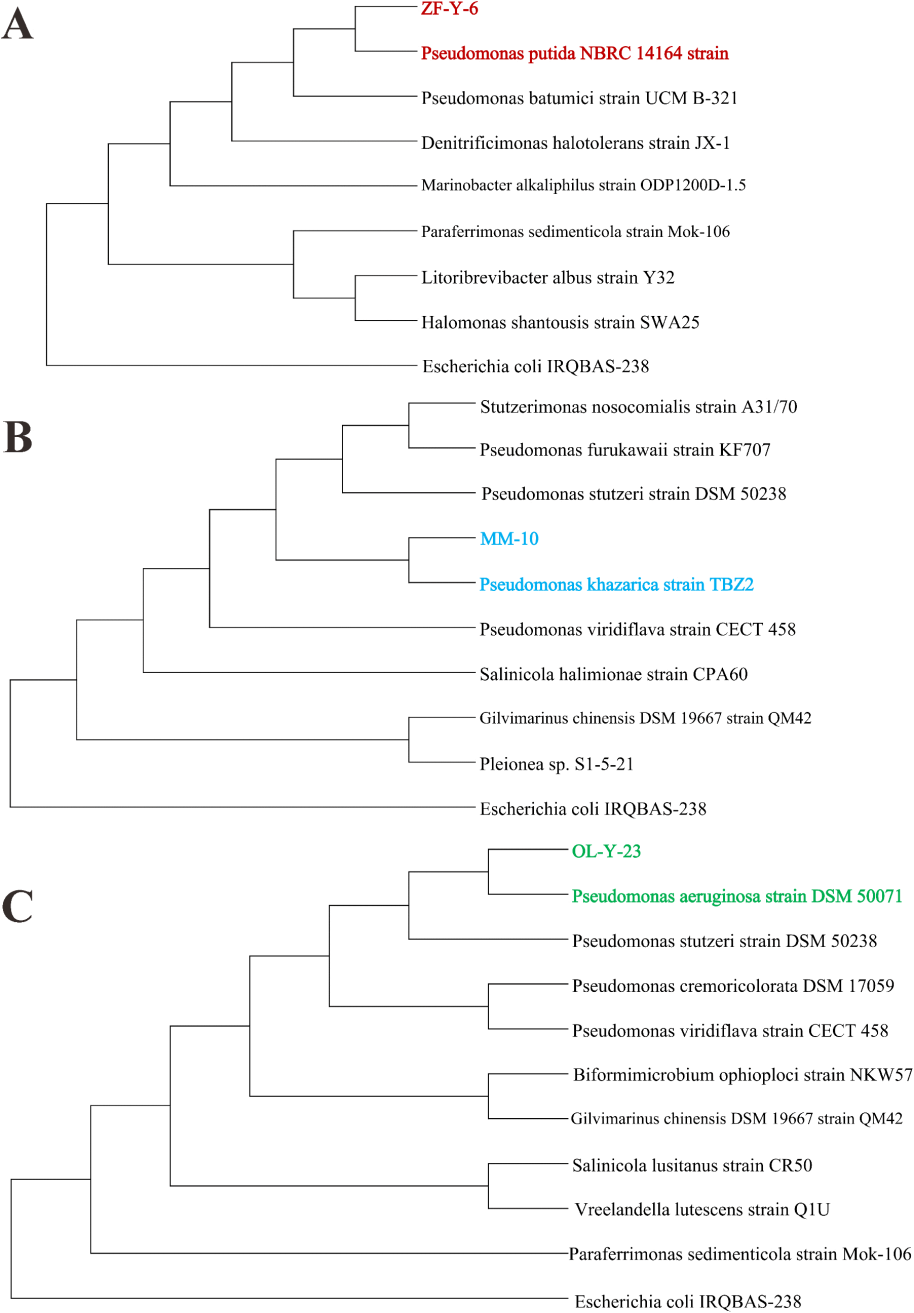
The evolutionary tree of identified fish intestinal bacterial strains. **(A)** The phylogenetic tree of ZF-Y-6 bacteria isolated from live zebrafish. **(B)** The phylogenetic tree of MM-10 bacteria isolated from marine medaka. **(C)** The phylogenetic tree of OL-Y-23 bacteria isolated from freshwater medaka model.

Among the above culturable strains, several bacteria with potential promising prospects were selected for the mapping of developmental trees, to explore the further interactions and functions in environment or host. In zebrafish, the strain of ZF-Y-6-live was grown in live zebrafish under anaerobic conditions, which showed most similar to *Pseudomonas* strains and then *Denitrificimonas*, *Marinobacter*, *Paraferrimonas*, and *Halomonas* bacteria (Fig 6A). In marine medaka, the strain of MM-10 was grown in seawater medaka dissected gut under aerobic conditions, and was discovered with highest similar to the *Pseudomonas* strains and then *Salinicola*, *Stutzerimonas*, *Gilvimarinus*, and *Pleionea* bacteria (Fig 6B). In freshwater medaka, the OL-Y-23 bacteria was isolated from the dissected gut under anaerobic conditions, which was also most similar to *Pseudomonas* strains, but then then *Biformimicrobium*, *Gilvimarinus*, *Salinicola*, *Vreelandella*, and *Paraferrimonas* bacteria (Fig 6C). These bacteria strains belonged to the same genus but from the three typical fish species, indicating the similar as well as different functions in corresponding host and living habit or surroundings.

### 3.7. Metabolic functions of fish intestinal bacteria detected by API 20E kits

**Fig 7:**
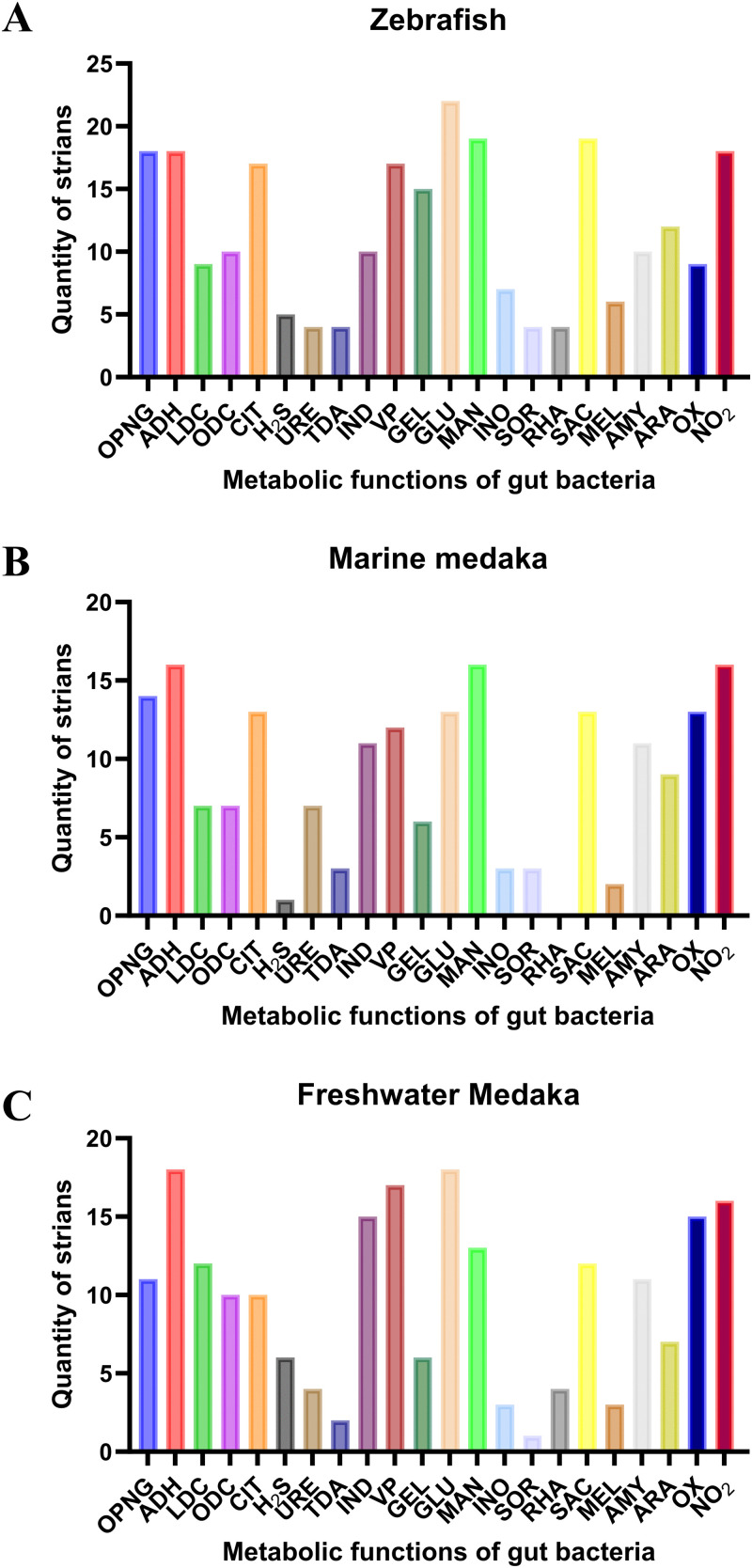
Analysis of metabolic functions of intestinal bacteria in different fish models. **(A)** The statistical analysis of metabolic functions of the intestinal bacterial strains isolated in zebrafish. **(B)** The general display of metabolic functions of the intestinal bacterial strains screened out in marine medaka. **(C)** It represents the metabolic functions of the intestinal bacterial strains identified in freshwater medaka by API 20E kits. Abbreviations: ONPG, o-nitrophenyl-β-D-galactopyranoside; ADH, arginine dihydrolase; LDC, lysine decarboxylase; ODC, ornithine decarboxylase; CIT, citrate utilization; H2S, hydrogen sulfide production; URE, urease; TDA, tryptophan deaminase; IND, indole production; VP, Voges–Proskauer reaction; GEL, gelatinase; GLU, glucose fermentation; MAN, mannitol; INO, inositol; SOR, sorbitol; RHA, rhamnose; SAC, sucrose; MEL, melibiose; AMY, amygdalin; ARA, arabinose; OX, cytochrome oxidase; NO2, nitrogen dioxide reduction.

Different fish models were necessarily explored to illustrate at adult stage, which potentially provide the understanding the effects and mechanisms of gut microbiota on host growth and development. In this study, the metabolic function of fish intestinal bacterial strains *in vitro* was detected by using the API 20E kits, and zebrafish intestinal strains showed generally metabolize OPNG, ADH, VP, GEL, GLU, MAN, SAC, and NO_2_. Marine medaka intestinal bacterial strains can usually metabolize ADH, MAN, and NO_2_, and freshwater medaka can normally metabolize ADH, IND, GLU, OX, and NO_2_ (Table 4). Among the screened zebrafish intestinal bacteria, 18 strains can decompose the OPNG, ADH, and NO_2_, 9 strains can decompose the LDC and OX, 10 strains can react on ODC, IND, and AMY substrate, and 17 strains metabolic the CIT and VP. Specially, there were 5 strains positively react on H_2_S, and a total of 4 strains induced URE, TDA, SOR, and RHA decomposed. Moreover, most of bacterial 15 strains can degrade the GEL (15 strains), GLU (22 strains), MAN and ASC (19 strains). And 7 strains decomposed the INO, 6 strains decomposed the MEL, and 12 strains degraded the ARA **(**Fig 7A**)**.

**Table 4:** The metabolic functions of identified bacterial strains from fish intestines.

	Name		Metabolic functions																					
**No.**	**Zebrafish-bacteria**	**Genus**	**ONPG**	**ADH**	**LDC**	**ODC**	**CIT**	**H**_**2**_ **S**	**URE**	**TDA**	**IND**	**VP**	**GEL**	**GLU**	**MAN**	**INO**	**SOR**	**RHA**	**SAC**	**MEL**	**AMY**	**ARA**	**OX**	**NO**_**2**_
**1**	**ZF-H-1**	***Pseudomonas***	**–**	**+**	**–**	**–**	**+**	**–**	**–**	**–**	**/**	**+**	**+**	**–**	**–**	**–**	**–**	**–**	**–**	**–**	**–**	**–**	**/**	**/**
**2**	**ZF-H-2**	***Aeromonas***	**+**	**+**	**+**	**–**	**+**	**–**	**–**	**+**	**/**	**+**	**+**	**+**	**+**	**–**	**–**	**–**	**+**	**–**	**–**	**–**	**/**	**/**
**3**	**ZF-H-3**	***Shewanella***	**–**	**+**	**–**	**–**	**+**	**+**	**–**	**–**	**/**	**–**	+*	**–**	**–**	**–**	**–**	**–**	**–**	**–**	**–**	**–**	**/**	**/**
**4**	**ZF-H-4**	***Vibrio***	**+**	**+**	**+**	**+**	**+**	**–**	**–**	**+**	**/**	**+**	**+**	**+**	**+**	**–**	**–**	**–**	**+**	**–**	**+**	**–**	**/**	**/**
**5**	**ZF-H-5**	***Microbacterium***	**+**	**–**	**–**	**–**	**+**	**–**	**–**	**–**	**/**	**+**	**+**	**+**	**–**	**–**	**–**	**–**	**–**	**–**	**+**	**–**	**/**	**/**
**6**	**ZF-H-6**	***Rhodococcus***	**–**	**–**	**–**	**–**	**+**	**–**	**+**	**–**	**/**	**+**	**–**	**–**	**–**	**–**	**–**	**–**	**–**	**–**	**–**	**–**	**/**	**/**
**7**	**ZF-H-7**	***Exiguobacterium***	**+**	**+**	**–**	**–**	**+**	**–**	**–**	**–**	**/**	**+**	+*	**+**	**+**	**–**	**+**	**–**	**+**	**+**	**+**	**+**	**/**	**/**
**8**	**ZF-H-8**	***Bacillus***	**+**	**–**	**–**	**–**	**+**	**–**	**–**	**–**	**/**	**+**	+*	**+**	**+**	**+**	**–**	**–**	**+**	**+**	**+**	**–**	**/**	**/**
**9**	**ZF-Y-1**	***Shewanella***	**–**	**+**	**–**	**+**	**–**	**+**	**–**	**–**	**–**	**–**	**+**	**–**	**–**	**–**	**–**	**–**	**–**	**–**	**–**	**+**	**+**	**+**
**10**	**ZF-Y-2**	***Vibrio***	**+**	**–**	**–**	**–**	**–**	**–**	**–**	**–**	**–**	**–**	**–**	**+**	**+**	**–**	**–**	**–**	**+**	**–**	**–**	**+**	**–**	**+**
**11**	**ZF-Y-3–7.6**	***Plesiomonas***	**+**	**+**	**+**	**+**	**+**	**–**	**–**	**–**	**+**	**–**	**–**	**+**	**+**	**+**	**–**	**–**	**–**	**–**	**–**	**–**	**+**	**+**
**12**	**ZF-Y-4**	***Pseudomonas***	**+**	**+**	**–**	**–**	**+**	**+**	**–**	**–**	**–**	**+**	**–**	**–**	**–**	**–**	**–**	**–**	**–**	**–**	**–**	**–**	**+**	**+**
**13**	**ZF-Y-5**	***Pseudomonas***	**–**	**+**	**+**	**+**	**+**	**+**	**–**	**+**	**+**	**+**	**–**	**+**	**+**	**–**	**–**	**–**	**+**	**–**	**+**	**–**	**+**	**+**
**14**	**ZF-Y-6–6.25**	***Vibrio***	**–**	**–**	**–**	**–**	**–**	**–**	**–**	**–**	**–**	**–**	**–**	**+**	**+**	**–**	**–**	**–**	**+**	**–**	**–**	**+**	**–**	**+**
**15**	**ZF-Y-8**	***Vibrio***	**+**	**+**	**+**	**+**	**+**	**–**	**–**	**–**	**+**	**–**	**+**	**+**	**+**	**–**	**+**	**–**	**+**	**–**	**+**	**+**	**+**	**+**
**16**	**ZF-Y-9**	***Bacillus***	**+**	**–**	**–**	**–**	**–**	**–**	**–**	**–**	**–**	**–**	**–**	**–**	**+**	**–**	**–**	**–**	**–**	**–**	**–**	**–**	**–**	**–**
**17**	**ZF-Y-13**	***Aeromonas***	**+**	**+**	**+**	**+**	**+**	**+**	**–**	**–**	**+**	**+**	**+**	**+**	**+**	**–**	**–**	**–**	**+**	**–**	**–**	**–**	**+**	**+**
**18**	**ZF-Y-15**	***Vibrio***	**+**	**–**	**–**	**–**	**–**	**–**	**–**	**+**	**+**	**–**	**+**	**+**	**+**	**+**	**+**	**+**	**+**	**–**	**–**	**+**	**–**	**+**
**19**	**ZF-X-1-live**	***Acinetobacter***	**–**	**+**	**+**	**+**	**+**	**–**	**+**	**–**	**–**	**+**	**+**	**+**	**–**	**–**	**–**	**+**	**+**	**+**	**–**	**+**	**–**	**–**
**20**	**ZF-X-3-live**	***Acinetobacter***	**+**	**+**	**–**	**–**	**+**	**–**	**+**	**–**	**–**	**+**	**+**	**+**	**+**	**+**	**–**	**–**	**+**	**–**	**+**	**–**	**–**	**–**
**21**	**ZF-X-15-live**	***Acinetobacter***	**–**	**–**	**–**	**–**	**–**	**–**	**–**	**–**	**–**	**+**	**–**	**+**	**–**	**–**	**–**	**+**	**–**	**+**	**–**	**+**	**–**	**–**
**22**	**ZF-H-4-live**	***Plesiomonas***	**+**	**+**	**+**	**+**	**–**	**–**	**–**	**–**	**+**	**–**	**–**	**+**	**–**	**+**	**–**	**–**	**–**	**+**	**–**	**–**	**–**	**+**
**23**	**ZF-Y-2-live**	***Rhizobium***	**–**	**–**	**–**	**–**	**–**	**–**	**–**	**–**	**–**	**+**	**+**	**–**	**+**	**+**	**–**	**–**	**–**	**–**	**–**	**–**	**+**	**+**
**24**	**ZF-Y-3-live**	***Acinetobacter***	**+**	**+**	**–**	**–**	**+**	**–**	**–**	**–**	**+**	**–**	**–**	**+**	**–**	**–**	**+**	**+**	**+**	**+**	**+**	**+**	**–**	**+**
**25**	**ZF-Y-4-live**	***Brevundimonas***	**–**	**+**	**–**	**–**	**–**	**–**	**–**	**–**	**–**	**+**	**+**	**+**	**+**	**–**	**–**	**–**	**+**	**–**	**+**	**–**	**+**	**+**
**26**	**ZF-Y-5-live**	***Staphylococcus***	**+**	**–**	**–**	**–**	**+**	**–**	**+**	**–**	**+**	**+**	**–**	**+**	**+**	**–**	**–**	**–**	**+**	**–**	**–**	**–**	**–**	**+**
**27**	**ZF-y-2**	***Vibrio***	**+**	**–**	**–**	**–**	**–**	**–**	**–**	**–**	**–**	**–**	**–**	**+**	**+**	**–**	**–**	**–**	**+**	**–**	**–**	**+**	**–**	**+**
**28**	**ZF-y-3**	***Plesiomonas***	**+**	**+**	**+**	**+**	**–**	**–**	**–**	**–**	**+**	**–**	**–**	**+**	**–**	**+**	**–**	**–**	**–**	**–**	**–**	**+**	**+**	**+**
**29**	**ZF-y-5**	***Staphylococcus***	**–**	**–**	**–**	**+**	**–**	**–**	**–**	**–**	**–**	**+**	**–**	**+**	**+**	**–**	**–**	**–**	**+**	**–**	**–**	**+**	**–**	**+**
**30**	**ZF-y-6**	***Janibacter***	**–**	**+**	**–**	**–**	**–**	**–**	**–**	**–**	**+**	**–**	**–**	**–**	**+**	**–**	**–**	**–**	**+**	**–**	**+**	**–**	**–**	**+**
**No.**	**Marine medaka-bacteria**	**Genus**	**ONPG**	**ADH**	**LDC**	**ODC**	**CIT**	**H**_**2**_ **S**	**URE**	**TDA**	**IND**	**VP**	**GEL**	**GLU**	**MAN**	**INO**	**SOR**	**RHA**	**SAC**	**MEL**	**AMY**	**ARA**	**OX**	**NO**_**2**_
**1**	**MM-H-1**	***Vibrio***	**+**	**+**	**+**	**+**	**–**	**–**	**–**	**–**	**+**	**–**	**+**	**+**	**+**	**–**	**–**	**–**	**+**	**+**	**+**	**–**	**+**	**+**
**2**	**MM-H-2**	***Vibrio***	**+**	**+**	**+**	**+**	**+**	**–**	**+**	**–**	**+**	**–**	**+**	**+**	**+**	**–**	**–**	**–**	**+**	**–**	**+**	**–**	**+**	**+**
**3**	**MM-H-6**	***Vibrio***	**–**	**–**	**–**	**–**	**+**	**–**	**–**	**–**	**+**	**–**	**–**	**+**	**+**	**–**	**–**	**–**	**+**	**–**	**+**	**–**	**–**	**+**
**4**	**MM-H-8**	***Pseudomonas***	**–**	**+**	**–**	**–**	**+**	**–**	**–**	**–**	**–**	**+**	**–**	**–**	**–**	**–**	**–**	**–**	**–**	**–**	**–**	**–**	**+**	**–**
**5**	**MM-H-10**	***Pseudomonas***	**–**	**+**	**–**	**–**	**+**	**–**	**–**	**–**	**–**	**+**	**–**	**–**	**+**	**–**	**–**	**–**	**–**	**–**	**–**	**–**	**+**	**–**
**6**	**MM-H-11**	***Staphylococcus***	**+**	**–**	**–**	**–**	**–**	**–**	**+**	**–**	**–**	**+**	**–**	**+**	**+**	**+**	**+**	**–**	**+**	**–**	**–**	**+**	**–**	**–**
**7**	**MM-H-19**	***Dietzia***	**–**	**–**	**–**	**–**	**–**	**–**	**–**	**–**	**–**	**+**	**–**	**–**	**–**	**–**	**–**	**–**	**–**	**–**	**–**	**–**	**–**	**+**
**8**	**MM-H-20**	***Exiguobacterium***	**+**	**–**	**–**	**–**	**–**	**–**	**–**	**–**	**–**	**–**	**–**	**–**	**+**	**–**	**–**	**–**	**–**	**–**	**–**	**–**	**–**	**–**
**9**	**MM-H-23**	***Shewanella***	**–**	**+**	**+**	**+**	**+**	**+**	**+**	**–**	**–**	**–**	**+**	**–**	**+**	**–**	**–**	**–**	**+**	**–**	**–**	**+**	**+**	**+**
**10**	**MM-Y-1**	***Vibrio***	**+**	**+**	**–**	**+**	**–**	**–**	**–**	**+**	**+**	**+**	**–**	**+**	**+**	**–**	**+**	**–**	**+**	**–**	**+**	**+**	**+**	**+**
**11**	**MM-Y-2**	***Vibrio***	**+**	**+**	**+**	**–**	**+**	**–**	**–**	**–**	**+**	**+**	**–**	**+**	**+**	**–**	**–**	**–**	**–**	**–**	**+**	**+**	**+**	**+**
**12**	**MM-Y-6**	***Vibrio***	**+**	**+**	**+**	**+**	**–**	**–**	**–**	**+**	**+**	**–**	**–**	**+**	**+**	**+**	**+**	**–**	**+**	**+**	**+**	**+**	**+**	**+**
**13**	**MM-Y-9**	***Vibrio***	**+**	**+**	**–**	**–**	**+**	**–**	**–**	**–**	**+**	**+**	**–**	**+**	**+**	**–**	**–**	**–**	**+**	**–**	**+**	**+**	**+**	**+**
**14**	**MM-Y-11**	***Vibrio***	**+**	**+**	**–**	**–**	**+**	**–**	**–**	**–**	**+**	**+**	**–**	**+**	**+**	**–**	**–**	**–**	**+**	**–**	**+**	**+**	**+**	**+**
**15**	**MM-Y-13**	***Vibrio***	**+**	**+**	**–**	**–**	**+**	**–**	**–**	**–**	**+**	**+**	**–**	**+**	**+**	**–**	**–**	**–**	**+**	**–**	**+**	**+**	**+**	**+**
**16**	**MM-Y-14**	***Bacillus***	**–**	**–**	**–**	**–**	**–**	**–**	**–**	**–**	**–**	**–**	**–**	**–**	**+**	**+**	**–**	**–**	**–**	**–**	**–**	**–**	**–**	**–**
**17**	**OM-y-1**	***Rhodococcus***	**+**	**–**	**–**	**–**	**–**	**–**	**+**	**–**	**+**	**–**	**–**	**+**	**–**	**–**	**–**	**–**	**+**	**–**	**+**	**–**	**–**	**+**
**18**	**OM-y-2**	***Brevundimonas***	**+**	**+**	**–**	**–**	**–**	**–**	**–**	**–**	**–**	**–**	**–**	**–**	**+**	**–**	**–**	**–**	**–**	**–**	**+**	**+**	**+**	**+**
**19**	**OM-y-3**	***Stenotrophomonas***	**–**	**+**	**–**	**–**	**+**	**–**	**–**	**–**	**+**	**–**	**–**	**+**	**+**	**–**	**–**	**–**	**–**	**–**	**–**	**–**	**–**	**+**
**20**	**OM-y-4**	***Staphylococcu***	**+**	**+**	**–**	**–**	**+**	**–**	**+**	**–**	**–**	**+**	**+**	**–**	**–**	**–**	**–**	**–**	**+**	**–**	**–**	**–**	**–**	**+**
**21**	**HH-1**	***Bacillus***	**–**	**+**	**+**	**+**	**+**	**–**	**+**	**–**	**–**	**+**	**+**	**+**	**–**	**–**	**–**	**–**	**+**	**–**	**–**	**–**	**+**	**–**
**22**	**HH-3**	***Deinococcus***	**+**	**–**	**–**	**–**	**–**	**–**	**–**	**+**	**–**	**+**	**+**	**/**	**–**	**–**	**–**	**–**	**–**	**–**	**–**	**–**	**–**	**+**
**23**	**HH-X-2**	***Acinetobacter***	**–**	**+**	**+**	**+**	**+**	**–**	**+**	**–**	**–**	**–**	**–**	**–**	**–**	**–**	**–**	**–**	**–**	**–**	**–**	**–**	**–**	**–**
**No.**	**Japanese medaka-bacteria**	**Genus**	**ONPG**	**ADH**	**LDC**	**ODC**	**CIT**	**H**_**2**_ **S**	**URE**	**TDA**	**IND**	**VP**	**GEL**	**GLU**	**MAN**	**INO**	**SOR**	**RHA**	**SAC**	**MEL**	**AMY**	**ARA**	**OX**	**NO**_**2**_
**1**	**OL-H-1**	***Aeromonas***	**+**	**+**	**+**	**+**	**+**	**–**	**–**	**–**	**+**	**+**	**–**	**+**	**–**	**–**	**–**	**+**	**+**	**–**	**+**	**–**	**+**	**+**
**2**	**OL-H-2**	***Vibrio***	**+**	**+**	**–**	**–**	**–**	**–**	**–**	**–**	**+**	**+**	**–**	**+**	**+**	**–**	**–**	**–**	**+**	**–**	**+**	**+**	**+**	**+**
**3**	**OL-H-6**	***Shewanella***	**–**	**+**	**+**	**+**	**–**	**+**	**–**	**–**	**+**	**–**	**–**	**+**	**–**	**–**	**–**	**+**	**+**	**–**	**–**	**+**	**+**	**–**
**4**	**OL-H-11**	***Flavobacterium***	**+**	**+**	**+**	**–**	**–**	**–**	**–**	**–**	**+**	**+**	**–**	**+**	**–**	**–**	**–**	**+**	**+**	**+**	**+**	**–**	**–**	**+**
**5**	**OL-H-14**	***Shewanella***	**–**	**+**	**+**	**+**	**–**	**+**	**–**	**–**	**+**	**–**	**–**	**+**	**–**	**–**	**–**	**+**	**+**	**–**	**–**	**–**	**+**	**–**
**6**	**OL-H-19**	***Brevundimonas***	**–**	**+**	**–**	**+**	**–**	**+**	**–**	**–**	**–**	**–**	**–**	**–**	**–**	**–**	**–**	**–**	**–**	**–**	**–**	**–**	**+**	**+**
**7**	**OL-H-20**	***Acinetobacter***	**–**	**–**	**–**	**–**	**–**	**–**	**–**	**–**	**–**	**+**	**–**	**–**	**–**	**–**	**–**	**–**	**–**	**–**	**–**	**–**	**–**	**–**
**8**	**OL-H-21**	***Acinetobacter***	**–**	**–**	**–**	**–**	**–**	**–**	**–**	**–**	**–**	**+**	**–**	**–**	**–**	**–**	**–**	**–**	**–**	**–**	**–**	**–**	**–**	**–**
**9**	**OL-H-23**	***Chryseobacterium***	**–**	**–**	**–**	**–**	**–**	**–**	**–**	**–**	**+**	**–**	**+**	**+**	**–**	**–**	**–**	**–**	**–**	**–**	**–**	**–**	**+**	**+**
**10**	**OL-H-24**	***Aeromonas***	**+**	**+**	**+**	**+**	**+**	**+**	**–**	**–**	**+**	**+**	**+**	**+**	**+**	**–**	**–**	**–**	**–**	**–**	**–**	**–**	**+**	**+**
**11**	**OL-Y-1**	***Shewanella***	**–**	**+**	**+**	**+**	**+**	**+**	**–**	**–**	**+**	**–**	**+**	**+**	**+**	**–**	**–**	**–**	**+**	**–**	**+**	**+**	**+**	**+**
**12**	**OL-Y-2**	***Aeromonas***	**+**	**+**	**+**	**–**	**+**	**–**	**–**	**–**	**+**	**+**	**+**	**+**	**+**	**–**	**–**	**–**	**+**	**–**	**+**	**–**	**+**	**+**
**13**	**OL-Y-3**	***Microbacterium***	**–**	**–**	**–**	**–**	**+**	**–**	**–**	**–**	**–**	**+**	**–**	**–**	**–**	**–**	**–**	**–**	**–**	**–**	**–**	**–**	**–**	**+**
**14**	**OL-Y-5**	***Microbacterium***	**–**	**–**	**–**	**–**	**–**	**–**	**–**	**–**	**–**	**+**	**–**	**+**	**+**	**+**	**+**	**–**	**+**	**+**	**+**	**+**	**–**	**–**
**15**	**OL-Y-6**	***Vibrio***	**+**	**+**	**+**	**+**	**+**	**+**	**–**	**–**	**+**	**+**	**–**	**+**	**+**	**+**	**–**	**–**	**+**	**–**	**+**	**+**	**+**	**+**
**16**	**OL-Y-9**	***Priestia***	**+**	**–**	**–**	**–**	**–**	**–**	**–**	**–**	**–**	**+**	**–**	**–**	**+**	**–**	**–**	**–**	**–**	**–**	**–**	**–**	**–**	**–**
**17**	**OL-Y-15**	***Cytobacillus***	**–**	**–**	**–**	**–**	**–**	**–**	**–**	**–**	**–**	**–**	**–**	**–**	**–**	**–**	**–**	**–**	**–**	**–**	**–**	**–**	**–**	**+**
**18**	**OL-Y-19**	***Microbacterium***	**+**	**–**	**–**	**–**	**–**	**–**	**–**	**–**	**–**	**+**	**–**	**–**	**–**	**–**	**–**	**–**	**–**	**–**	**–**	**–**	**–**	**–**
**19**	**OL-Y-20**	***Flavobacterium***	**+**	**+**	**+**	**–**	**–**	**–**	**–**	**–**	**+**	**+**	**–**	**+**	**+**	**–**	**–**	**–**	**–**	**–**	**+**	**–**	**–**	**+**
**20**	**OL-Y-21**	***Brevibacterium***	**–**	**–**	**–**	**–**	**–**	**–**	**–**	**–**	**–**	**+**	**–**	**–**	**–**	**–**	**–**	**–**	**–**	**–**	**–**	**–**	**+**	**–**
**21**	**OL-Y-22**	***Pseudomonas***	**+**	**+**	**+**	**+**	**+**	**–**	**–**	**–**	**+**	**–**	**–**	**+**	**+**	**+**	**–**	**–**	**+**	**–**	**+**	**+**	**+**	**–**
**22**	**OL-Y-23**	***Pseudomonas***	**–**	**+**	**–**	**–**	**+**	**–**	**–**	**–**	**–**	**–**	**+**	**+**	**+**	**–**	**–**	**–**	**–**	**+**	**–**	**+**	**+**	**+**
**23**	**OL-y-1**	***Staphylococcus***	**+**	**+**	**–**	**–**	**–**	**–**	**+**	**–**	**+**	**+**	**–**	**+**	**+**	**–**	**–**	**–**	**+**	**–**	**–**	**–**	**–**	**–**
**24**	**OL-y-2**	***Corynebacterium***	**–**	**–**	**–**	**–**	**–**	**–**	**–**	**–**	**+**	**+**	**–**	**+**	**+**	**–**	**–**	**–**	**–**	**–**	**+**	**–**	**–**	**–**
**25**	**OL-y-3**	***Rhodococcus***	**–**	**+**	**–**	**–**	**–**	**–**	**+**	**–**	**–**	**–**	**–**	**+**	**+**	**–**	**–**	**–**	**+**	**–**	**–**	**–**	**–**	**+**
**26**	**DH-1**	***Bacillus***	**–**	**+**	**–**	**–**	**+**	**–**	**–**	**+**	**–**	**+**	**+**	**+**	**–**	**–**	**–**	**–**	**–**	**–**	**+**	**–**	**–**	**+**
**27**	**DH-3**	***Vogesella***	**–**	**+**	**+**	**+**	**–**	**–**	**+**	**+**	**+**	**–**	**–**	**–**	**–**	**–**	**–**	**–**	**–**	**–**	**–**	**–**	**+**	**–**
**28**	**DY-4**	***Aquipseudomonas***	**–**	**+**	**+**	**+**	**+**	**–**	**+**	**–**	**–**	**–**	**–**	**–**	**–**	**–**	**–**	**–**	**–**	**–**	**–**	**–**	**+**	**+**

Note: 1. The first 8 genus were detection in our previous report [Jia-2019] and summarized in the Table 2.The obvious positive phenomena of this assay appeared at 19–24 h, and the “+” means positive, the “-” means negative to metabolize respective substance, “/” means no detections. 3.Totally 22 tests included o-Nitrophenyl-β-D-Galactopyranoside (ONPG), arginine dihydrolase (ADH), lysine decarboxylase (LDC), ornithine decarboxylase (ODC), citrate (CIT), hydrogen sulfide (H_2_S), urease (URE), tryptophane deaminase (TDA), Indole test (IND), Voges Proskauer (VP), gelatinase (GEL), glucose (GLU), mannitol (MAN), inositol (INO), sorbitol (SOR), rhamnose (RHA), saccharose (SAC), melibiose (MEL), amygdalin (AMY), and arabinose (ARA), cytochrome oxidase (OX), and nitrogen dioxide (NO_2_)

In terms of metabolic functions of isolated intestinal strains in marine medaka (Table 4), totally 14 strains can decompose the OPNG, 16 strains can decompose the ADH, NO_2_ and MAN, 7 strains decomposed the LDC, ODC, and URE, and 13 strains degraded the CIT, GLU, SAC, and OX. Interestingly, only 1 strain positively react on H_2_S, and 3 strains can decompose TDA, INO, and SOR, but there were 11 strains that showed the IND and AMY positive reaction. For the special items, totally 12 strains can decompose the VP, and 6 strains related to GEL, 2 strains related to MEL, and 9 strains metabolized the ARA (Fig 7B).

Among the isolated intestinal bacteria of freshwater medaka, totally 11 strains can break down the OPNG and AMY, and 18 strains decomposed the ADH and GLU. Moreover, most bacteria can metabolize the LDC and SAC (12 strains), ODC and CIT (10 strains), IND and OX (15 strains), VP (17 strains), MAN (13 strains), and NO_2_ (16 strains). Additionally, there were 6 strains that can decompose the H_2_S and GEL, and 4 strains related to URE and RHA, 7 strains related to INO and ARA. Specially, only 2 or 3 strains reacted on TDA or INO or MEL, and 1 strain decomposed SOR (Fig 7C). Therefore, the detection demonstrated that these intestinal strains respectively have their own various metabolic functions, indicating the different and important roles in host. For example, *Vibrio* specifically metabolized ONPG, ADH, CIT, IND, GLU, SAC, AMY, NO_2_ and OX. *Pseudomonas* specifically metabolized ADH, CIT, VP, and OX. *Staphylococcus* specifically metabolized ONPG, URE, VP, GLU, SAC, and ARA. *Exiguobacterium* specifically metabolized ONPG and SOR (Table 4).

## 4. Discussion

Zebrafish and medaka are widely used fish models for studies of host–microbiota interactions, because their gut microbial communities are relatively complex and vary with host development and environmental background [10, 36]. In the present study, the natural gut microbiota of zebrafish, marine medaka, and freshwater medaka showed clear differences in bacterial diversity and richness, indicating that host species and living environment jointly shape intestinal microbial communities. At the same time, the culture-derived bacterial collections recovered from these three fish models also differed markedly among species and cultivation conditions, suggesting that both biological background and experimental workflow influenced the final isolate library.

For zebrafish, previous studies have shown that gut microbial communities become more complex with host development and are affected by host age, immune status, and environmental conditions [37]. Consistent with these findings, our results showed that the isolated culturable bacteria from the adult zebrafish under aerobic and anaerobic conditions according to morphological identification were mainly belonged to the phyla of Proteobacteria, Pseudomonadota, Actinobacteria, Bacillota, Firmicutes, and Actinomycetota. Compared with 16S rRNA gene-based sequencing and NCBI–BLAST search against various databases, it was found that the original intestinal microbiota of zebrafish mainly included the phyla of Pseudomonadota, Firmicutes, Bacteroidetes, Actinobacteria, and Acidobacteria, etc. This comparison indicated that the isolate collection reflected part, but not all, of the natural gut microbial composition. Therefore, the zebrafish bacterial library established here should be regarded as a representative but incomplete subset of the original intestinal microbiota, which nonetheless provides useful material for downstream functional studies. The ecological relevance of microbiota composition is further supported by zebrafish studies showing that community structure can influence biologically meaningful host outcomes, such as colonization resistance against pathogen infection [38].

Marine medaka and freshwater medaka further highlighted the effects of habitat background on gut bacterial composition. The ocean environment and pollution toxicity have increasingly attracted attentions in recent years, and the development and application of marine medaka as powerful animal models are urgently needed. In this study, the diversity and richness of gut microbiota showed that the dominant phyla were Proteobacteria, Verrucomicrobia, Actinobacteria, Firmicutes, Bacteroidetes, and Cyanobacteria, most of which were decreased in both female and male marine medaka during growth. Furthermore, the dominant changed genera were *Vibrio*, while *Bacillus*, *Pseudomonas*, *Staphylococcus*, *Stenotrophomonas*, *Dietzia* and so on. In the marine and freshwater medaka, diverse culturable bacteria were identified as the genera of *Vibrio*, *Bacillus*, *Pseudomonas*, *Dietzia*, *Rhodococcus*, *Acinetobacter*, *Shewanella*, and *Microbacterium*. Therefore, findings in this work furtherly revealed the distribution, analysis, and comparison of gut microbial composition and culturable library in classical fish models, which may act as the important factors to evaluate the underlying contribution to fish health, growth, and functional changes.

Previous studies have reported that the culturable gut microbiota of fish is mainly composed of recurrent genera such as Aeromonas, Vibrio, Pseudomonas, and Staphylococcus [18]. Culture-based studies in marine and freshwater fish have also shown that combining phenotypic characterization with bacterial isolation can provide useful resources for understanding microbiota composition and for identifying strains with potential probiotic functions [27,39]. However, more comprehensive bacterial libraries established under both aerobic and anaerobic conditions, together with preliminary functional characterization of the recovered isolates, are still needed. For the significance of gut bacterial library, the potential benefits included the improve immunity, metabolite production, and antibiotic resistant, especially providing more resources for the probiotics’ exploration [ 40–42]. In this study, the culturable intestinal bacteria in zebrafish, marine and freshwater medaka were detected most with Gram-negative stain, such as *Pseudomonas*, *Aeromonas*, *Shewanella*, *Vibrio*, *Microbacterium*, and *Rhodococcus*, *Dietzia*, and a part strains of genera like *Exiguobacterium*, *Bacillus Staphylococcus*, and *Acinetobacter* showed Gram-positive stain. More importantly, their common functions identified to generally metabolize OPNG, ADH, CIT, AMY, IND, VP, GEL, GLU, MAN, SAC, OX, and NO_2_, and specially SOR and MEL were discovered in zebrafish, and H_2_S and RHA in freshwater medaka, indicating the different roles of each bacterium in host. Considering these promising metabolic functions, the isolated strains in this study will support the further investigation to determine their probiotic effects *in vitro* and in fish.

The functional relevance of fish gut bacteria has been increasingly recognized in recent years. In terms of the application of culturable gut microbiota, numerous aspects or research fields urgently need the isolated and identified single pure strain, and suitable and powerful animal models. Due to the absence of a microbiome and the defects in digestive and immune systems, studies on GF fish models have been limited to the early life stages in which depended on the yolk energy supply without eating, such as in GF zebrafish with innate immunity before 7 dpf [43]. Marine medaka was considered as the potential marine fish model for studying immunity, with the development of immune organs and innate and adaptive immune defense from the larval stage [44,45]. In fish, the larval and juvenile to adult period are normally characterized by high energetic requirements to support high growth rates and striking transformations of tissues and organs [46]. However, it was reported that in GF zebrafish and rodent animal models, absence of the microbiota reduced intestinal cell proliferation, including the goblet cells and associated immune cells, and disrupted metabolism and innate immunity, which suggesting the effects of the gut microbiota on host organs, especially on intestinal development [13,15,47]. All these studies showed the significant defect during the growth, indicating that absence of the gut microbiota in fish may contribute to a deficiency in intestinal system development and weak digestive or absorption ability. Furthermore, in fish, the goblet cells which distributed among the epithelial cells of the intestinal and respiratory tracts, can secrete mucus, a viscous fluid composed primarily of highly glycosylated proteins called mucins [23,48,49]. Mucus layers serve important functions in intestinal metabolic and inflammation processes, including in protection against shear stress and chemical damage, and trapping and elimination of particulate microorganisms [50–52]. A previous study also reported that the gut microbiota was closely related to host reproduction and that probiotic regulation positively influenced the physiological performance, including the development and reproduction of zebrafish models [53]. John *et al*. reported the generation of GF zebrafish and their application in studying the effects of the microbiota on epithelial renewal and enterocyte morphology, as well as the host transcriptional responses to the microbiota in larval models [54]. Recently, a study demonstrated the probiotic characteristics of the *Bacillus subtilis* isolated from rohu fish (*Labeo rohita*) by inhibiting *Aeromonas hydrophila* and *Edwardsiella tarda* using *in vitro* conditions and *in vivo* gnotobiotic zebrafish gut, which indicated its ability as an effective alternative to antibiotics in aquaculture [55]. Therefore, we suspected that with the application GF models of both zebrafish and medaka [56], it can be discovered the possibly imperfect functions such as distinct developmental delay by the absence of gut microbiota or colonization of special bacterial strains.

Several of the cultured isolates obtained in this study may also have practical application potential. In live zebrafish, the strain of ZF-Y-6-live was cultivated under anaerobic conditions, as a species of *Pseudomonas putida*, which may provide assistance in many aspects of human life. This bacterium has a certain biological control potential, and can inhibit the mycelial growth of *Fusarium oxysporum f. sp. lycopersici*, the main pathogen causing Fusarium wilt of tomato, and also promote the growth of tomatoes [57]. Clinically, this bacterium also has certain functions, for *Pseudomonas putida* strain NBRC 14164, which is most similar to ZF-Y-6-live, can efficiently obtain iron ions in an iron-deficient environment by secreting iron vectors (such as pyoverdine) or by utilizing the receptor-mediated iron transport system, demonstrating significant iron metabolic adaptability [58]. In the marine medaka, the strain of MM-10 was isolated and cultured under aerobic conditions in the intestinal exploration, with the most similar strain of *Pseudomonas khazarica* strain TBZ2 as the first new species of PaHs-degrading pseudomonas isolated from Caspian sea sediments, which was confirmed to have the potential for efficient degradation of polycyclic aromatic hydrocarbons (PAHs) [35]. While in freshwater medaka, the OL-Y-23 was isolated by intestinal samples cultured under anaerobic conditions, and its most similar strain *Pseudomonas aeruginosa* strain DSM 50071 could efficiently degrade polystyrene by serine hydrolase [59]. Additionally, a previous study proved that the isolation of *Bacillus thuringiensis* bacterium from the gut of freshwater fish, *Systomus sarana*, which can be used to produce maximum protease enzyme, and to elucidate of peptide profile of the protease [60]. However, it was worth noting that whether and how these strains initially isolated have potentials to benefit to environmental and host health, especially the regulate functions and mechanism, should be revealed *in vivo* and *in vitro* before application in bio-medical fields in the future.

In the present study, the *Shewanella oneidensis* strain MR-1 was screened and cultured from all of species (zebrafish, marine medaka and freshwater medaka), which has development potential for utilization in all aspects of human life. Because of extensive sulfonamides application in aquaculture and animal husbandry and the consequent increase in sulfonamides discharged into the environment, strategies to remediate sulfonamide-contaminated environments are essential. A previous study also proved that *Shewanella oneidensis* strain MR-1 was potential bacterial resource for biodegrading sulfonamides and therefore may bioremediate of sulfonamide-polluted environments [61]. Also, it can biodegrade hexabromocyclododecane, a widely used brominated flame retardant, under certain conditions, opening the way for environmentally friendly applications [62]. However, except the *Shewanella* strains, more investigation of the isolated culturable bacteria from fish models should be implemented to obtain the well understanding of the interaction and mechanism of “bacteria-bacteria” and “bacteria-host” in the future. For another example, three different *Bacillus* strains isolated from zebrafish, *Anguilla japonica* (Japanese eel), and hybrid sturgeon (*Acipenser baerii*♀ × *Acipenser schrenckii*♂) showed the ability as probiotics to against *Aeromonas* infection, effectively reduce harmful bacteria, and improve gut microbiota composition, and enhance digestive functions [63–65]. In addition, based on the library of both the gut microbiota and the phages in host [66], it will be significant and promising to develop the powerful strategies or techniques, probiotics or “bio-markers” for preventing bacteria-related human diseases by precisely regulation *in vitro* and *in vivo*.

At the same time, several limitations should be noted. First, bacterial isolation in this study relied mainly on general rich media, which improved isolation efficiency and ensured reproducibility across batches, but may have introduced culture-dependent selection bias by favoring fast-growing, aerotolerant, or facultatively anaerobic bacteria. As a result, strictly anaerobic taxa and gut commensals with specific nutritional requirements may be underrepresented, and the isolate collection should be interpreted as a partial rather than exhaustive representation of the gut microbiota. This bias may also explain why some important genera reported previously in zebrafish, such as Cetobacterium, were not recovered under the conditions used here. Future studies should expand the cultivation workflow by incorporating a broader and more targeted panel of media, together with stricter anaerobic cultivation strategies and parallel cultivation under diverse conditions, to improve the recovery and representativeness of key gut-associated taxa. Second, taxonomic identification in this study was mainly based on 16S rRNA gene sequencing. Although blast similarity can reliably support genus-level assignment and, in some cases, species-level inference when sequence identity is sufficiently high, its taxonomic resolution is limited for closely related taxa. Therefore, these findings should not be interpreted as definitive species-level identification without complementary evidence, such as additional molecular markers, whole-genome approaches, or more comprehensive phenotypic characterization. Third, phenotypic characterization was primarily based on the API 20E system, which is convenient and standardized but mainly oriented toward Enterobacteriaceae and other common Gram-negative bacteria and may have limited discriminatory power for non-enteric, fastidious, or anaerobic taxa; moreover, other API panels, particularly anaerobe-oriented systems, were not systematically evaluated. Therefore, the phenotypic profiles reported here should be considered preliminary biochemical evidence rather than definitive identification, and future work should combine complementary phenotypic assays with molecular and genome-based approaches to improve taxonomic resolution and functional characterization of the recovered isolates. Finally, conventional monoculture-based isolation may not efficiently recover gut bacteria that depend on metabolic cooperation or shared growth factors. Reduced-complexity microbial consortia may therefore serve as a valuable complementary approach for improving the cultivation of such taxa. Future work combining pure-culture isolation with consortium-based enrichment strategies may help increase isolation success and better capture the diversity of fish gut microbiota.

In summary, this study provided a comparative culture-based overview of the gut bacteria of zebrafish, marine medaka, and freshwater medaka and established the library of isolated community. Despite the inherent limitations of cultivation-based approaches, the recovered isolates can provide important resource for future studies on host-microbiota interactions, probiotic development, and functional validation.

## 5. Conclusion

In conclusion, this study explored the isolated library of gut microbiota in fish models involving zebrafish, marine and freshwater medaka, with the identification of characteristics and gram positive or negative of bacterial strains. Importantly, the innovative approaches to gain samples from fish intestines, as well as the diverse culture conditions were displayed in the protocol, which contributed to the scope of culturable bacteria *in vitro*. Moreover, the present study discovered multiple aerobic and anaerobic strains from typical three fish models with the detections of metabolic functions, which can benefit to investigate the inner mechanisms between the special bacteria and host health. Taken together, all these findings provide the primary microbial resource with sequencing and metabolic functions background, novel insights, and important potentials for application combined with diverse GF fish models in biomedicine, probiotic agent, and drug research regions.

## Supporting information

S1 Text The 16S sequencing information of zebrafish gut bacteria (ZF-live-H-1). (PDF)

S1 Fig The detection of isolated bacterial PCR production by 1% Gel electrophoresis (zebrafish was taken as the example). (TIF)

S2 Fig The sequencing peaks of isolated gut bacteria in zebrafish (The example was ZF-live-H-1. (TIF)
