# Supplementary material for: The library of isolated bacteria from gut microbiota in classical fish models: Zebrafish (Danio rerio), marine (Oryzias melastigma) and freshwater (Oryzias latipes) medaka
Source: PLoS One. 2026 May 15;21(5):e0347661. doi: 10.1371/journal.pone.0347661 (PMC13178932; doi:10.1371/journal.pone.0347661)
Supplement: S1 Text — (PDF) [file pone.0347661.s001.pdf]

Text S1. The 16S sequencing information of zebrafish gut bacteria (ZF-live-H-1) was as follows:

GGGCATGGCGGCAGCTTACACATGCAGTCGAGCGGGCGAAGGTTAGCCTTTCGCGTACACTGA

CCTAGCGGCGGACGGGTGAGTAATGCTTAGGAATCTGCCTATTAGTGGGGGACAACATTCCGAA

AGGAATGCTAATACCGCATACGCCCTACGGGGGAAAGCAGGGGATCTTCGGACCTTGCGCTAAT

AGATGAGCCTAAGTCAGATTAGCTAGTTGGTGGGGTAAAGGCCTACCAAGGCGACGATCTGTAG

CGGGTCTGAGAGGATGATCCGCCACACTGGGACTGAGACACGGCCCAGACTCCTACGGGAGGC

AGCAGTGGGGAATATTGGACAATGGGCGCAAGCCTGATCCAGCCATGCCGCGTGTGTGAAGAA

GGCCTTTTGGTTGTAAAGCACTTTAAGCGAGGAGGAGGCTACTTGGATTAATACTCTAGGATAG

TGGACGTTACTCGCAGAATAAGCACCGGCTAACTCTGTGCCAGCAGCCGCGGTAATACAGAGG

GTGCGAGCGTTAATCGGATTTACTGGGCGTAAAGCGTGCGTAGGCGGCTTTTTAAGTCGGATGT

GAAATCCCTGAGCTTAACTTAGGAATTGCATTCGATACTGGGAAGCTAGAGTATGGGAGAGGAT

GGTAGAATTCCAGGTGTAGCGGTGAAATGCGTAGAGATCTGGAGGAATACCGATGGCGAAGGC

AGCCATCTGGCCTAATACTGACGCTGAGGTACGAAAGCATGGGGAGCAAACAGGATTAGATACC

CTGGTAGTCCATGCCGTAAACGATGTCTACTAGCCGTTGGGGCCTTTGAGGCTTTAGTGGCGCA

GCTAACGCGATAAGTAGACCGCCTGGGGAGTACGGTCGCAAGACTAAAACTCAAATGAATTGAC

GGGGGCCCGCACAAGCGGTGGAGCATGTGGTTTAATTCGATGCAACGCGAAGAACCTTACCTG

GTCTTGACATAGTAAGAACTTTCCAGAGATGGATTGGTGCCTTCGGGAACTTACATACAGGTGC

TGCATGGCTGTCGTCAGCTCGTGTCGTGAGATGTTGGGTTAAGTCCCGCAACGAGCGCAACCC

TTTTCCTTATTTGCCAGCGGGTTAAGCCGGGAACTTTAAGGATACTGCCAGTGACAAACTGGAG

GAAGGCGGGGACGACGTCAAGTCATCATGGCCCTTACGACCAGGGCTACACACGTGCTACAAT

GGTCGGTACAAAGGGTTGCTACCTAGCGATAGGATGCTAATCTCAAAAAGCCGATCGTAGTCCG

GATTGGAGTCTGCAACTCGACTCCATGAAGTCGGAATCGCTAGTAATCGCGGATCAGAATGCCG

CGGTGAATACGTTCCCGGGCCTTGTACACACCGCCCGTCACACCATGGGAATTTGTTGCACCAG

AAGTAGGTAGTCTAACCGCAAGGAGGACGCTACCACGGTTCCGATTGTCT
